# Tumor microenvironment affects exogenous sodium/iodide symporter expression

**DOI:** 10.1016/j.tranon.2020.100937

**Published:** 2020-11-17

**Authors:** Fabio Castillo-Rivera, Alejandro Ondo-Méndez, Julien Guglielmi, Jean-Marie Guigonis, Lun Jing, Sabine Lindenthal, Andrea Gonzalez, Diana López, Béatrice Cambien, Thierry Pourcher

**Affiliations:** a Clinical Research Group, School of Medicine and Health Sciences, Universidad del Rosario, Bogota DC, Colombia; b Transporters in Imaging and Radiotherapy in Oncology (TIRO), School of Medicine, Direction de la Recherche Fondamentale (DRF), Institut des sciences du vivant Fréderic Joliot, Commissariat à l'Energie Atomique et aux énergies alternatives (CEA), Université Côte d'Azur (UCA), 28 Avenue de Valombrose, 06107 Nice, France; c Centro de Bioinformática y Biología Computacional de Colombia-BIOS, Manizales, Colombia; d Department of Biological Science, Faculty of Agricultural Sciences, Universidad Nacional de Colombia, Sede Palmira, Palmira, Colombia

**Keywords:** Sodium/Iodide symporter (NIS), Tumor microenvironment, Quiescence, Hypoxia, Protein trafficking

## Abstract

For decades, sodium/iodide symporter NIS-mediated iodide uptake has played a crucial role in the radioactive ablation of thyroid cancer cells. NIS-based gene therapy has also become a promising tool for the treatment of tumors of extrathyroidal origin. But its applicability has been hampered by reduced expression of NIS, resulting in a moderated capacity to accumulate ^131^I and in inefficient ablation. Despite numerous preclinical enhancement strategies, the understanding of NIS expression within tumors remains limited.

This study aims at a better understanding of the functional behavior of exogenous NIS expression in the context of malignant solid tumors that are characterized by rapid growth with an insufficient vasculature, leading to hypoxia and quiescence. Using subcutaneous HT29NIS and K7M2NIS tumors, we show that NIS-mediated uptake and NIS expression at the plasma membrane of cancer cells are impaired in the intratumoral regions. For a better understanding of the underlying molecular mechanisms induced by hypoxia and quiescence (separately and in combination), we performed experiments on HT29NIS cancer cells. Hypoxia and quiescence were both found to impair NIS-mediated uptake through mechanisms including NIS mis-localization. Modifications in the expression of proteins and metabolites involved in plasma membrane localization and in energy metabolism were found using untargeted proteomics and metabolomics approaches.

In conclusion, our results provide evidence that hypoxia and quiescence impair NIS expression at the plasma membrane, and iodide uptake. Our study also shows that the tumor microenvironment is an important parameter for successful NIS-based cancer treatment.

## Highlights

• NIS-expressing xenografts show low iodide uptake in areas exhibiting hypoperfusion.
• NIS subcellular localization and NIS expression is impaired under quiescent and/or hypoxic states.
• Proteomics and metabolomics reveal tumor microenvironment-triggered changes in proteins trafficking.
• Controlling microenvironmental factors is important for the efficacy of radioiodine therapy.

## Introduction

The sodium/iodide symporter (NIS) is a glycoprotein that is located at the basolateral membrane of thyrocytes and that mediates the active transport of iodide for thyroid hormone synthesis [[1], [2]–3]. For decades, NIS-mediated iodide accumulation in the thyrocytes has been a useful tool for the diagnosis and treatment of thyroid cancer [[4], [5], [6]]. It is well established that thyroid tumor cells mediate reduced iodide uptake [7], allowing the use of several gamma emitter substrates of NIS (^123^I and ^99m^Tc) for the diagnosis of thyroid tumor cells by scintigraphy, i.e. of malignant cold nodules. However, thyroid carcinomas are mainly differentiated (DTC), with preserved NIS expression and iodine uptake ability [8]. After total thyroidectomy, the remaining NIS expression then allows for radioiodine ablation of metastases and residual thyroid tissue using ^131^I.

After the identification of the NIS-encoding genes [9–11], iodide uptake could be evoked in non-thyroid cancer cells using gene therapy approaches. The resulting induced iodide uptake enabled noninvasive imaging of gene transfer monitoring and radioiodine ablation of targeted cells [12,13,14]. However, while promising results were obtained in preclinical trials on multiple myelomas [15, 16] and on hepatic carcinomas [17], other attempts failed to yield sufficient radioactive uptake for complete tumor cell ablation when applied to *in vivo* tumor models, despite the eradication of the same cell lines *in vitro* [[18], [19], [20]–21]. Clinical trials confirmed that the applicability of radioiodine to treat non-thyroidal neoplasms was hampered by the heterogenous NIS expression within lesions, resulting in a reduced capacity of such tumors to accumulate ^131^I [22, 23]. In this context, one should note that most of these *in vivo* assays were based on the use of subcutaneous xenograft tumors obtained after injection of cell lines, the predominant mouse solid tumor model [24].

In order to achieve efficient ablation using the maximal tolerable radioiodide activity, several strategies have been explored to improve NIS expression, NIS-mediated radioactive uptake and radiobiological effects.

For more than a decade now, research has been undertaken to unravel the post-transcriptional regulation of NIS [25], [26], [27], [28], [29]. Together with other groups, we have studied the higher iodide uptake ability of the rodent NIS compared to that of the human protein [30, 31], as well as the key molecular events involved in the post-translational regulation of NIS that could improve its localization at the plasma membrane [1,6,32,33]. To date, however, the underlying molecular mechanisms remain incompletely understood and transferring the rodent gene into patients may trigger deleterious immune responses. In this context, we recently provided evidence that achieving more uniform radioisotope ablation within xenografts using nanomedicine may help overcome resistance and reduce administered activities of radioactive iodine [34]. Our data, however, failed to address how tumor characteristics, in particular the tumor microenvironment, could influence the level of membranous NIS expression.

Attempts to increase radioactive retention by co-expression of NIS and exogenous thyroperoxidase [35,36,37] have been implemented, but they have produced insufficient synergistic benefits. Enhancement strategies were also developed to increase the dose *in situ* using either radioactive substrates with higher destructive potential such as the alpha-particle emitter ^211^At [38,39,40] and the beta-emitting radiometal ^188^Re [41,42,43] or by various radio-sensitizing approaches [34,44,45]. Improving exogenous NIS expression within non-thyroidal neoplasms has also been largely explored through direct protein delivery [46] and various gene transfer strategies. Among these are viruses [1, 47,48], nonviral gene delivery vectors [49,50] and more recently, stem cell-mediated gene delivery approaches [1, [51], [52], [53], [54]]. However, so far, despite encouraging results, such strategies still have to overcome practical limitations to their efficacy and clinical translation.

Most of the studies were carried out on both cultured cells and xenograft models using the same cell lines. As of today, few studies have been performed to evaluate the relevance of preclinical models and the use of subcutaneous xenograft mouse models in particular. However, using a hypoxia-responsive promoter and mesenchymal stem cell targeting, Muller and collaborators found significant tumor growth inhibition after application of a therapeutic dose of radioiodine in an orthotopic model, but not in a subcutaneous model [51].

In order to gain a deeper understanding of how the tumor microenvironment may influence NIS subcellular localization and uptake ability within tumors, we combined microSPECT/CT imaging and immunohistochemistry on models of rapidly-growing malignant solid tumors in which NIS was stably inserted. Our results show that both uptake and NIS expression are heterogeneously localized in HT29NIS xenografts, with higher expression observed in the proliferative and vascularized regions. To get a comprehensive overview of the effects of the microenvironment on NIS expression and localization, post-translational NIS regulation and metabolic changes within tumor cells, we performed radioiodine uptake, immunofluorescence, proteomics and metabolomics analyses on tumor cells cultured in conditions evoking hypoxia, normoxia, proliferation or quiescence. These observations may provide the basis for an improved understanding of the underlying molecular mechanisms.

## Materials and methods

### Cell culture

HT29, K7M2, and DHD cells were grown in Dulbecco´s Modified Eagle´s medium (DMEM, Gibco 31966-021) supplemented with 10% Fetal Bovine Serum (FBS, PAA, Gold A11-151), and 0.01 mg/ml penicillin/streptomycin (Gibco, Thermo Fischer Scientific, Courtaboeuf, France).

### NIS-expressing cell lines

NIS-expressing cell lines were established after transfection of human colon carcinoma HT29 cells (ATCC HTB-38, LGC Standards, Molsheim, France) and of murine osteosarcoma K7M2 cells (ATCC CRL‐2836) with the eukaryotic expression vector pcDNA3.1-mNIS [10]. Stable clones (HT29NIS) were selected as described [55].

### Animal studies

Animal housing and procedures were in accordance with the guidelines of the French Agriculture Ministry and were approved by the local ethics committee (Ciepal NCE/2015-223). For the induction of tumors, HT29NIS cells (2 x 10^6^ cells) or K7M2NIS cells were subcutaneously injected (5 × 10^5^ cells) in the back of 7-week-old SCID mice (Harlan, Gannat, France). SPECT imaging and immunohistochemistry experiments were performed when the tumors reached a diameter between 0.5 and 1 cm.

### In vivo SPECT/CT imaging

Animals were administered activities of 20MBq ^99m^TcO4^−^ intraperitoneally. Images of the tumor uptake were obtained using a dedicated microSPECT/CT scanner (eXplore speCZT CT120, General Electric) as previously described [55]. Reconstructed images were analyzed and quantified using AMIDE software. Uptakes were expressed as percentages of the injected activity after decay correction.

### Immunohistochemistry

Formalin-fixed, paraffin-embedded tumor tissue sections were stained using a rabbit polyclonal anti-NIS antibody (antibody 25, see [56]) followed by HRP-conjugated anti-rabbit antibodies and a 3,39–diaminobenzidine (DAB) co-substrate. The sections were counterstained with Harris hematoxylin (Sigma-Aldrich) before image acquisition.

### *In vitro* proliferation assays

Proliferating cells (cells with a normal rate of cell division) were obtained by seeding 2 × 10^6^ HT29 or HT29NIS cells in low confluence (10%) in DMEM supplemented with 10% FBS for 24 h. Upon reaching 50% confluence, cells were processed for the experiment. Quiescent cells (non-proliferative cells in reversible-rest state) were obtained by seeding 10 × 10^6^ HT29 or HT29NIS cells (at 50% confluence) in DMEM medium supplemented with 0.1% FBS for 48 h [57]_._ Cells were grown under hypoxic conditions (1% oxygen for 24 h in hypoxia chamber 200 INVIVO2 Hypoxia Workstation (Ruskinn Technology Ltd, UK)) or normoxia (21% oxygen).

### Cell viability analysis

Cell viability was assessed using the trypan blue dye exclusion test; cells were stained and counted as described [58].

### Iodide uptake assays

Iodide uptake in whole cells was performed according to [31] with 30 μM NaI and ^125^I. Measurements were made after 1 h in the presence or the absence of 100 mM perchlorate, a NIS inhibitor. The radioactivity was measured using a gamma counter (BioTraces, Herndon, Virginia, USA).

### NIS protein expression analysis

For Western blot analysis, tumor cell membrane proteins were subjected to electrophoresis as previously described [31]. Immunodetection was performed with anti-mNIS antibody 25 or with an anti-β-actin antibody (Sigma), followed by anti-rabbit or anti-mouse secondary antibodies, respectively (references 31460 or 31430, Thermo Fisher Scientific).

### Immunofluorescence

Tumor cells plated in 24‐well format were grown for 24 h in different growth conditions (normoxic or hypoxic; proliferative or quiescent) before being fixed (4% PFA and 0.02% glutaraldehyde) and permeabilized (0.1% triton). After a blocking step (BSA 1%), immunofluorescence labelling was performed using anti-mNIS antibody 25 as previously described [31]. Nuclei were stained with Hoechst 33342. Fluorescence images were captured and analyzed using a Cytation 3 imaging reader (BioTek instruments, Inc.).

### Proteomic analysis

Proteins from the cancer cells were extracted as previously described for Western blot analyses. Methods were as reported previously (59) and are detailed in the supplementary information.

### Metabolomics analysis

Plated cells were rinsed twice with a cold sucrose solution (150 mM), and cold methanol was added to each well. Plates were incubated overnight at –20 °C. After centrifugation at 15000 g for 15 min, supernatants were removed and dried using a SpeedVac concentrator. Metabolic profiling was performed using LC-MS/MS as previously described [59]. Detailed procedures are provided in supplementary information.

### Statistical analysis

For the iodide uptake assay and NIS protein expression, the results shown are the average ± SD (*n* = 3). One-factor ANOVA followed by the Posthoc–Bonferroni method were applied for all comparisons, as appropriate. *p* <0.05 was considered as significant. For proteomics and metabolomics, all experiments were performed in four independent replicates. Proteins and metabolites between groups were considered significantly different when *p* ≤ 0.05, up-regulated when fold change ≥2 and down-regulated when fold change ≤ 0.5.

## Results

### NIS-mediated uptake and NIS expression are not homogeneous in tumor xenografts

**Fig. 1:**
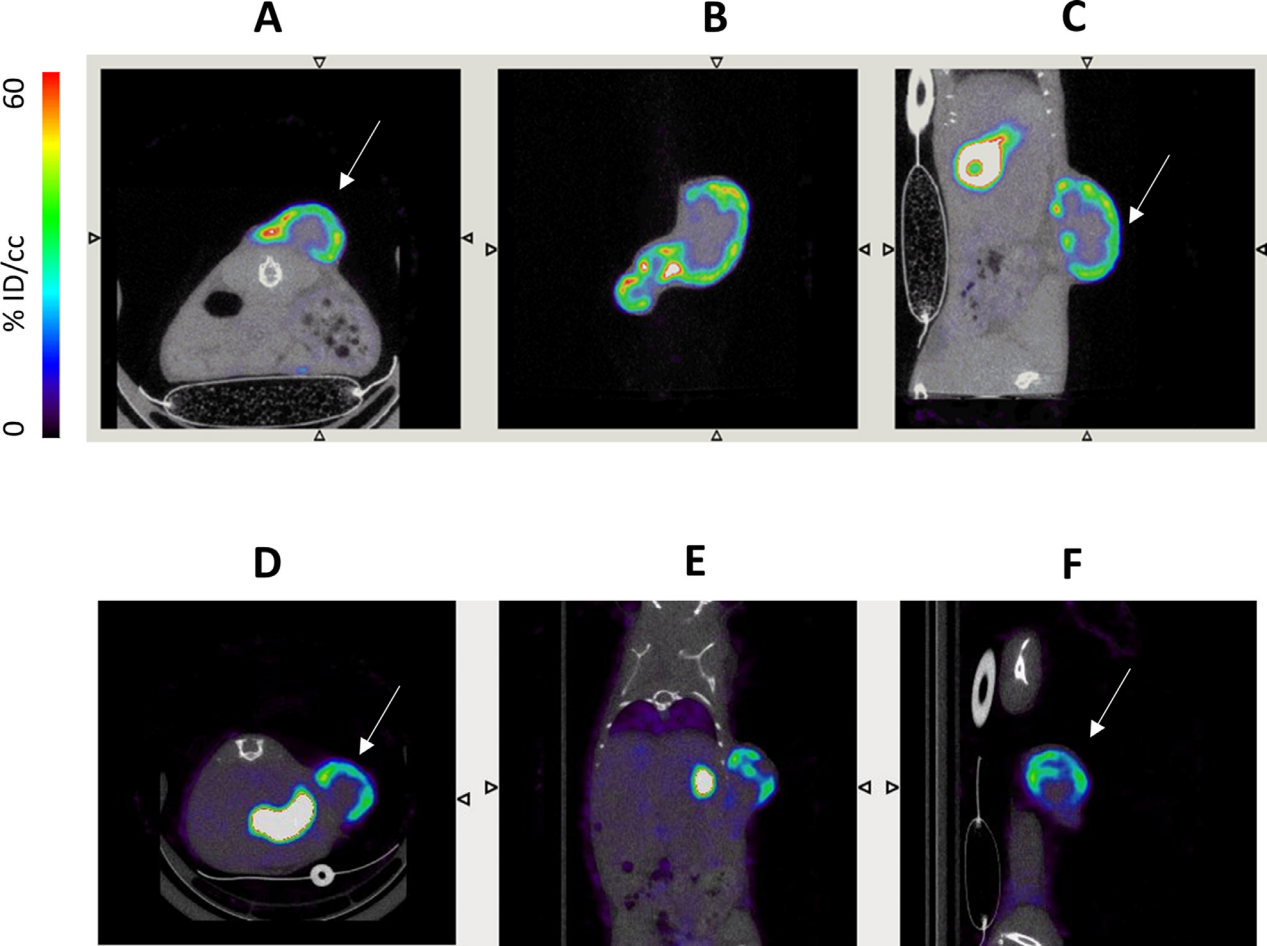
^99m^Tc pertechnetate uptake ability of subcutaneous HT29NIS and K7M2NIS tumors. Human colon cancer HT29NIS cells or murine osteosarcoma K7M2NIS cells expressing NIS were subcutanously inoculated into nude mice NOD SCID. Twenty-seven days after inoculation, serial SPECT/CT scans were performed. Mice received an intraperitoneal injection of 100 MBq ^99m^TcO_4_ and, 60 minutes after, mice were imaged with a microSPECT/CT camera (eXplore speCZT, General Electric). Transverse (A, D), coronal (B, E) and sagittal (C, F) slices were obtained from fused SPECT/CT images of the tumor area.

As shown in the representative images in Fig. 1(A)–(C), micro-SPECT imaging clearly showed that 99mTc pertechnetate uptake was mainly located at the external border of the HT29NIS tumors. Such a pattern was observed in all HT29NIS tumors with a diameter above 5 mm. A similar pattern of uptake was observed in tumor xenografts developed from other cell lines stably expressing exogenous NIS, murine osteosarcoma K7M2NIS cells (Fig. 1(D)–(F)) or rat colon cancer DHDNIS cells (data not shown).

**Fig. 2:**
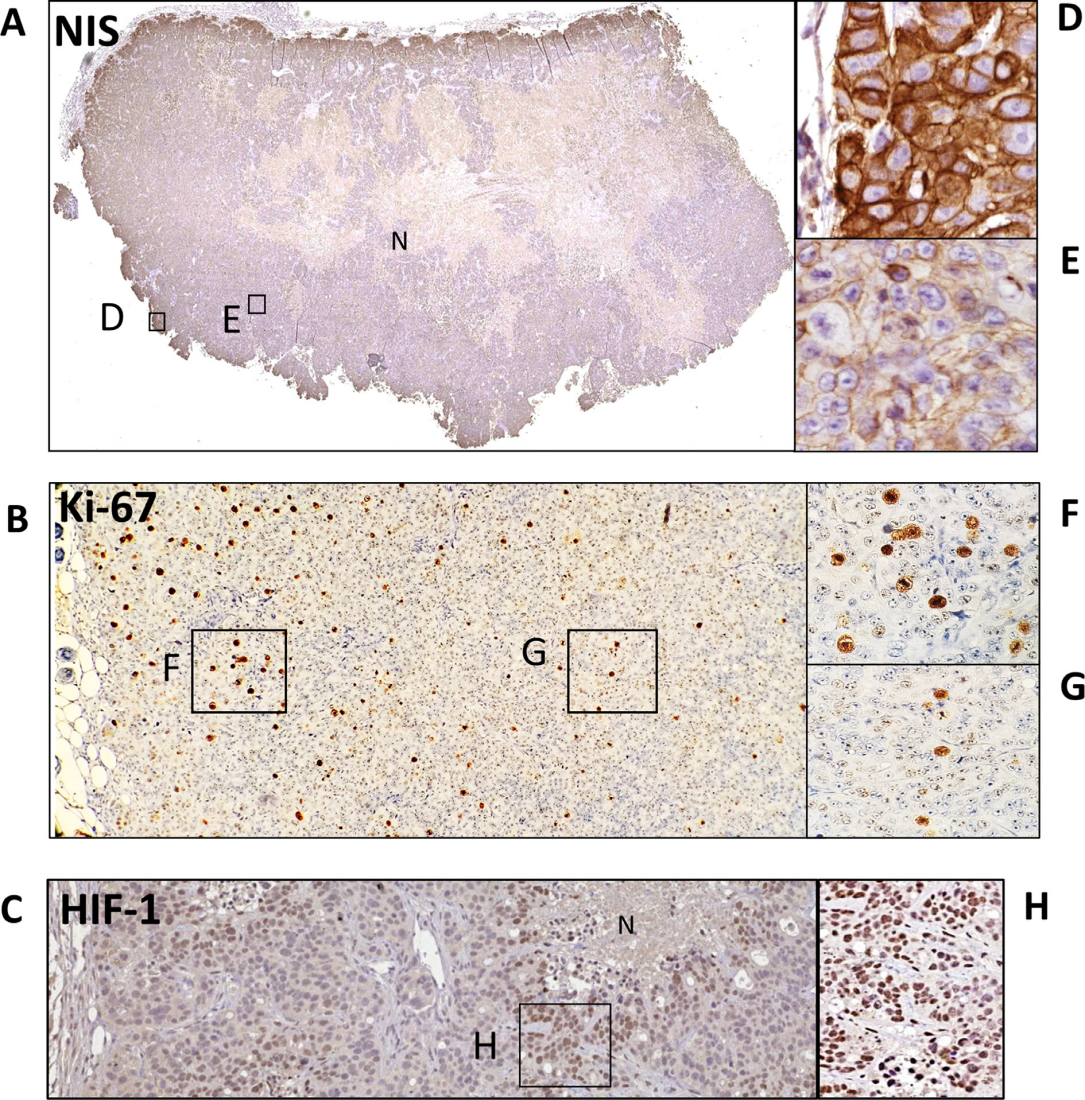
Representative immunohistochemistry detection of the NIS protein, Ki-67 and HIF-1alpha in subcutaneous HT29-NIS tumors. Colon cancer cells expressing NIS (2 × 10^6^) were subcutaneously inoculated into NOD SCID nude mice. Mice were sacrificed twenty-seven days after inoculation. (A) NIS immunostaining displays higher protein expression in the border of the tumor (D) and weaker and more diffuse expression in the more internal regions (E), no expression within necrotic areas (N). (B) Representative images of Ki-67 staining showing peripheral proliferative activity within HT29NIS (F) and low mitotic activity in internal areas (G). Higher HIF-1 immunostaining was observed in internal regions of the tumor surrounding necrotic areas, (H).

Interestingly, the pertechnetate uptake observed within peripheral areas of subcutaneous tumors in vivo (Fig. 1) was correlated with NIS expression as determined by immunohistochemistry. As illustrated in Fig. 2(A), stronger NIS immunostaining was observed at the plasma membrane of the cancer cells that are located at the border of the tumors (Fig. 2(D)) and that exhibit higher mitotic activity (Ki-67) (Fig. 2(B) and (F)). A weaker and more diffuse NIS expression was observed in the internal areas of the tumors (ratio=1.61, p <0.005, supplementary Fig. 1(A)), associated with lower percentages of Ki-67–positive tumor cells (Fig. 2(G)) (2.38% versus 18.61% for the internal and external areas, respectively p<0.001, supplementary Fig. (1B)). In the necrotic central areas of the tumors, NIS staining was lost (Fig. 2(A)). Peripheral proliferative areas were further confirmed by higher expression levels of carbonic anhydrase IX (CAIX) (supplementary Figure 1C). CAIX expression was associated with proliferative HT29 cells in a study using similar xenografts [60]. Internal areas of the tumor showing a weak NIS staining also displayed a strong HIF-1 immunostaining, suggesting hypoxic zones (Fig. 2(C) and (H)). Taken together, our results indicate that a weaker and more diffuse NIS expression in the internal parts of the tumor is associated with cancer cells exhibiting a lower proliferative status in a more hypoxic environment.

The vascularity and the perfusion of subcutaneous HT29-NIS tumors were also studied using ultrasounds. As shown in a representative illustration in Supplementary Fig. 2, tumors exhibited marked heterogeneity in blood perfusion, ranging from no or low perfusion in the center of the tumors to an increased perfusion towards the tumor periphery.

These results indicate that reductions in ^99m^Tc pertechnetate uptake and NIS expression in xenografts could be associated with tumor regions exhibiting reduced blood perfusion and proliferation, evoking hypoxic and quiescent tumor microenvironments.

### NIS-mediated iodide uptake is decreased by hypoxia and quiescence in cultured HT29NIS cells

We also studied NIS expression in cultured colorectal cancer cells grown in conditions mimicking hypoxia and quiescence.

Hypoxia of cultured cells was induced after incubation for 24 h in the presence of 1% oxygen in a dedicated incubator (control conditions are in the presence of 21% oxygen). Quiescent cells were induced by higher cell confluence combined with a reduced percentage (0.1%) of fetal bovine serum (FBS) in the culture media. As a control, proliferative cells were at low confluency (∼10%) and the media contained 10% FBS. In HT29NIS cells, a significative decrease (approximatively 20 percent) in cell viability was induced by quiescence and hypoxia (see Supplementary Fig. 3). In contrast, under similar culture conditions, the parental cell line HT29 (that does not express NIS) did not exhibit any decrease in cell viability. We also verified that a quiescent state was induced in our experimental conditions by measuring the expression of p27 protein, a negative regulator of cell cycle progression at G1. This protein is present at higher levels in quiescent cells and it is reduced in cells in a proliferative state (see Supplementary Fig. 4). In addition, results from our metabolomics experiments (see final results section) further demonstrated that our experimental conditions were mimicking hypoxia and quiescence. We verified that a hypoxic state was evoked in our experimental conditions by analyzing two proteins, Pyruvate Kinase M2 (PKM2) and Lactate Dehydrogenase A (LDHA), s that are up regulated by hypoxic conditions (Supplementary Fig. 5).

**Fig. 3:**
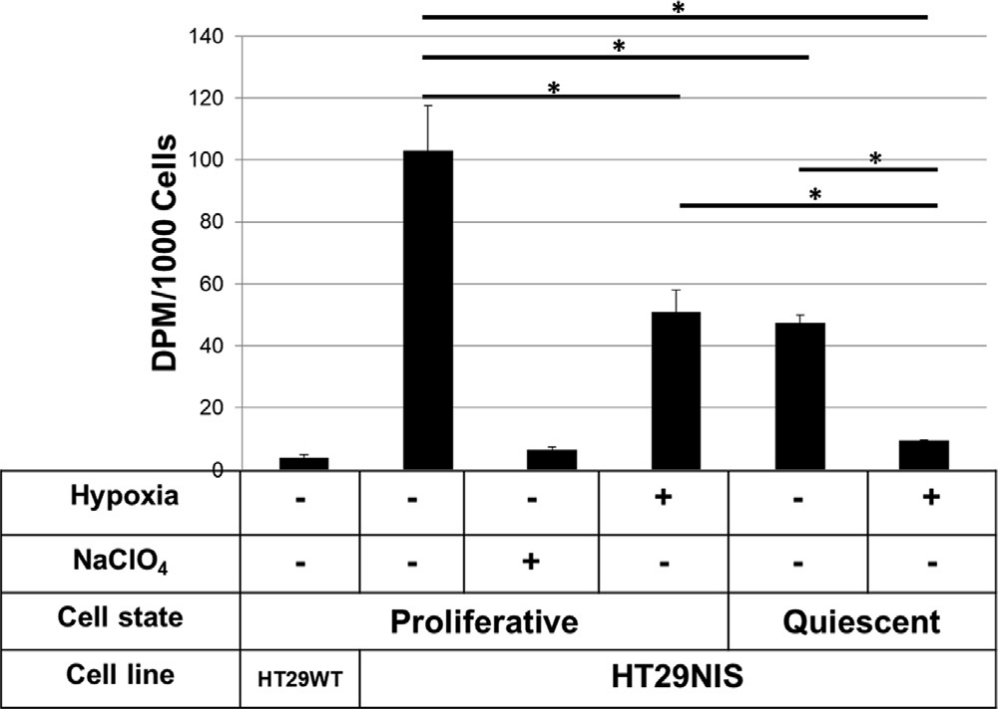
Iodide uptake in proliferative and quiescent HT29-NIS cells under normoxic and hypoxic conditions. The HT29WT cells (parental HT29 cells that do not express NIS) were used as a negative control and perchlorate (NaClO_4_) as a competitive inhibitor of NIS-mediated iodide transport. The results are shown as average ± SD of triplicates of 3 independent experiments. * p <0.05.

NIS-mediated iodide uptake assays were performed in proliferative and quiescent HT29NIS cells under normoxic and hypoxic conditions. As shown in Fig. 3, hypoxic conditions induced a significant reduction (50%±4) in NIS-mediated iodide uptake in proliferative cells. In addition, quiescence also significantly reduced (54%±2) NIS-mediated iodide uptake in normoxic conditions. Finally, iodide uptake was markedly decreased (91%±1) in quiescent cells under hypoxic conditions. In conclusion, our results indicate that hypoxia and quiescence induce cumulative impairments of NIS-mediated iodide uptake in HT29NIS cells.

### Subcellular localization of the NIS protein is altered in hypoxic and quiescent conditions

To further assess the mechanisms underlying the reduction in NIS uptake induced by hypoxia and quiescence, NIS protein expression, mRNA levels and subcellular localization were studied.

Immunoblotting and quantitative-RT-PCR experiments were performed on proliferative and quiescent HT29NIS cells cultured under normoxic and hypoxic conditions. As illustrated in Supplemental Fig. 6(A), NIS expression by proliferative HT29NIS cells was reduced (1.5-fold) in hypoxic conditions compared to normoxic conditions. A similar decrease induced by hypoxic conditions was observed (1.75-fold) in quiescent cells. Quantitative-RT-PCR results also showed a 2-fold reduction in mRNA levels induced by hypoxic conditions in both quiescent and proliferative cancer cells (Supplemental Fig. 6(B)). Our results indicate that hypoxia but not quiescence induces a decrease of NIS-protein expression in HT29NIS cells.

**Fig. 4:**
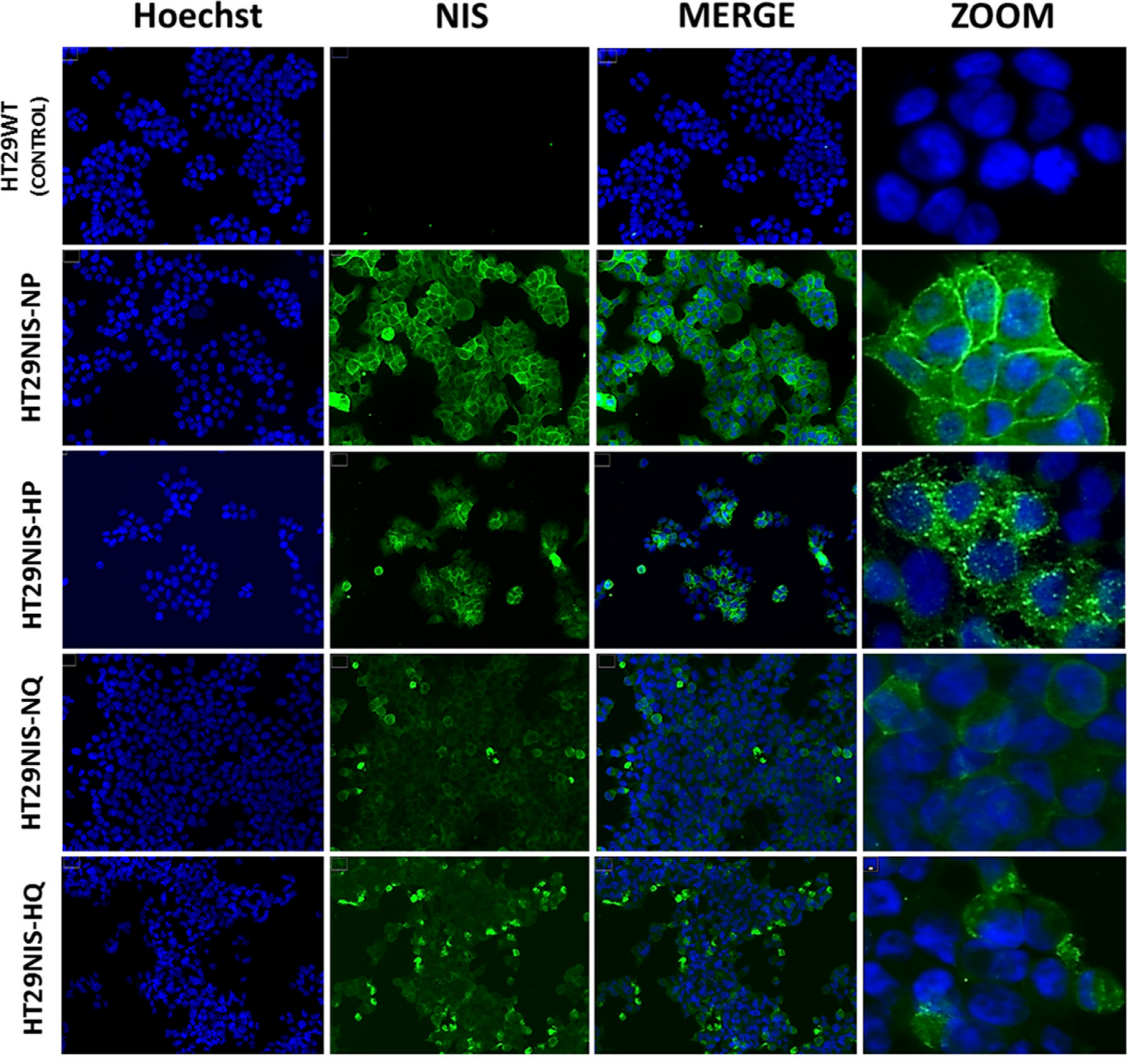
Subcellular localization of NIS protein by immunofluorescence in proliferative HT29NIS cells in normoxic conditions (HT29NIS-NP), proliferative HT29NIS cells in hypoxic conditions (HT29NIS-HP), quiescent HT29NIS cells in normoxic conditions (HT29NIS-QP) and quiescent HT29NIS cells in hypoxic conditions (HT29NIS-HQ). As a negative control wild type HT29 cells (HT29WT) are also shown.

NIS subcellular localization was compared in proliferative and quiescent HT29NIS cells under normoxic and hypoxic conditions. Representative immunocytochemistry analyses are shown in Fig. 4. In proliferative HT29NIS cells cultured under normoxic conditions (HT29NIS-NP), NIS expression appeared to be mainly located at the plasma membrane (see zoomed image). In contrast, NIS staining in proliferative HT29NIS cells under hypoxic conditions (HT29NIS-HP), was predominantly located in the cytosol and accumulated in intracellular areas. In quiescent HT29NIS cells under normoxic conditions (HT29NIS-NQ), NIS-specific immunostaining was dispersed in the cytosol of the cells and plasma membrane staining was rarely visible. Finally, for quiescent HT29NIS cells in hypoxic conditions (HT29NIS-HQ), NIS protein appeared both diffuse in the cytosol and accumulated in intracellular areas. NIS-specific immunostaining was virtually absent at the plasma membrane. In conclusion, our results indicate that hypoxia and quiescence impair NIS-protein expression at the plasma membrane of HT29NIS cells and induce distinct patterns of NIS subcellular localization, suggesting that the underlying mechanisms that drive changes in localization are diverse.

### Hypoxic and quiescent conditions induce proteome modifications

**Fig. 5:**
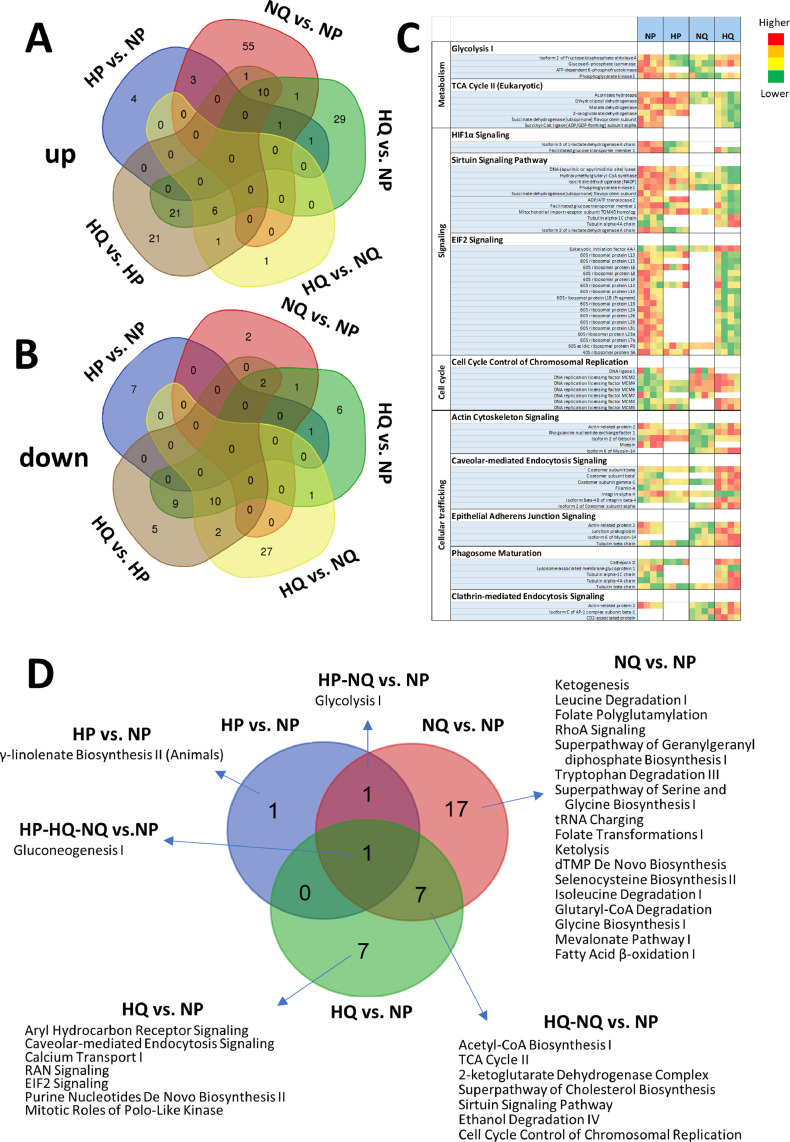
Variation in protein levels of proliferative HT29NIS cells in normoxic conditions (NP), proliferative HT29NIS cells in hypoxic conditions (HP), quiescent HT29NIS cells in normoxic conditions (QP) and quiescent HT29NIS cells in hypoxic conditions (HQ). (A) Venn diagram of significantly up-regulated and (B) down-regulated proteins. (C) Heat map of the significantly changed protein levels related to selected pathways. Green indicates higher levels and red indicates lower levels. (D) Venn diagram of significantly altered pathways. Only significantly altered pathways compared to NP are shown. A value of p <0.05 was considered significant.

The molecular mechanisms underlying NIS retention induced by hypoxia and quiescence were assessed by proteomics experiments on proliferative and quiescent HT29NIS cells cultured under normoxic or hypoxic conditions. Variations of protein expression and related pathways are shown in Supplementary Data 1 and Supplementary Fig. 7. As shown in the Venn diagram in Fig. 5(A), most of the significant protein upregulations are related to the quiescence state, but they differ according to hypoxic or normoxic conditions. The Venn diagram in Fig. 5(B) shows that significatively downregulated proteins were mainly found when hypoxic quiescent cells were compared to normoxic quiescent cells. The heatmap in Fig. 5(C) illustrates the significant variations in the expression level of the proteins involved in relevant pathways. Glycolysis-related protein levels were increased in proliferative cells under hypoxic conditions and in quiescent cells under normoxic conditions. As expected from hypoxic conditions, HIF1 signaling was significantly induced in proliferative cells under hypoxic conditions (Fig. 5(C) and supplementary data 1). Also, as expected, the levels of the proteins of the cell cycle pathway were reduced in quiescent cells (Fig. 5(C) and supplementary data 1). In addition, levels of TriCarboxylic Acid (TCA)-cycle-related proteins were increased in quiescent cells. For HIF1α, sirtuin and EIF2 signaling, most of the related proteins showed higher levels in quiescent cells in hypoxic conditions. As expected, lower levels of cell-cycle-related proteins were linked to quiescence. Cellular trafficking, actin-cytoskeleton-related proteins were found to be upregulated in quiescent cells under normoxic conditions. However, for Calveolar-mediated-endocytosis, epithelial-adherens-junction, phagosome-maturation and clathrin-mediated-endocytosis pathways, related proteins showed reduced expression in quiescent cells under hypoxic conditions. The Venn diagram in Fig. 5(D) summarizes all the significantly- impacted pathways.

### Hypoxic and quiescent conditions induce modifications of the metabolome

**Fig. 6:**
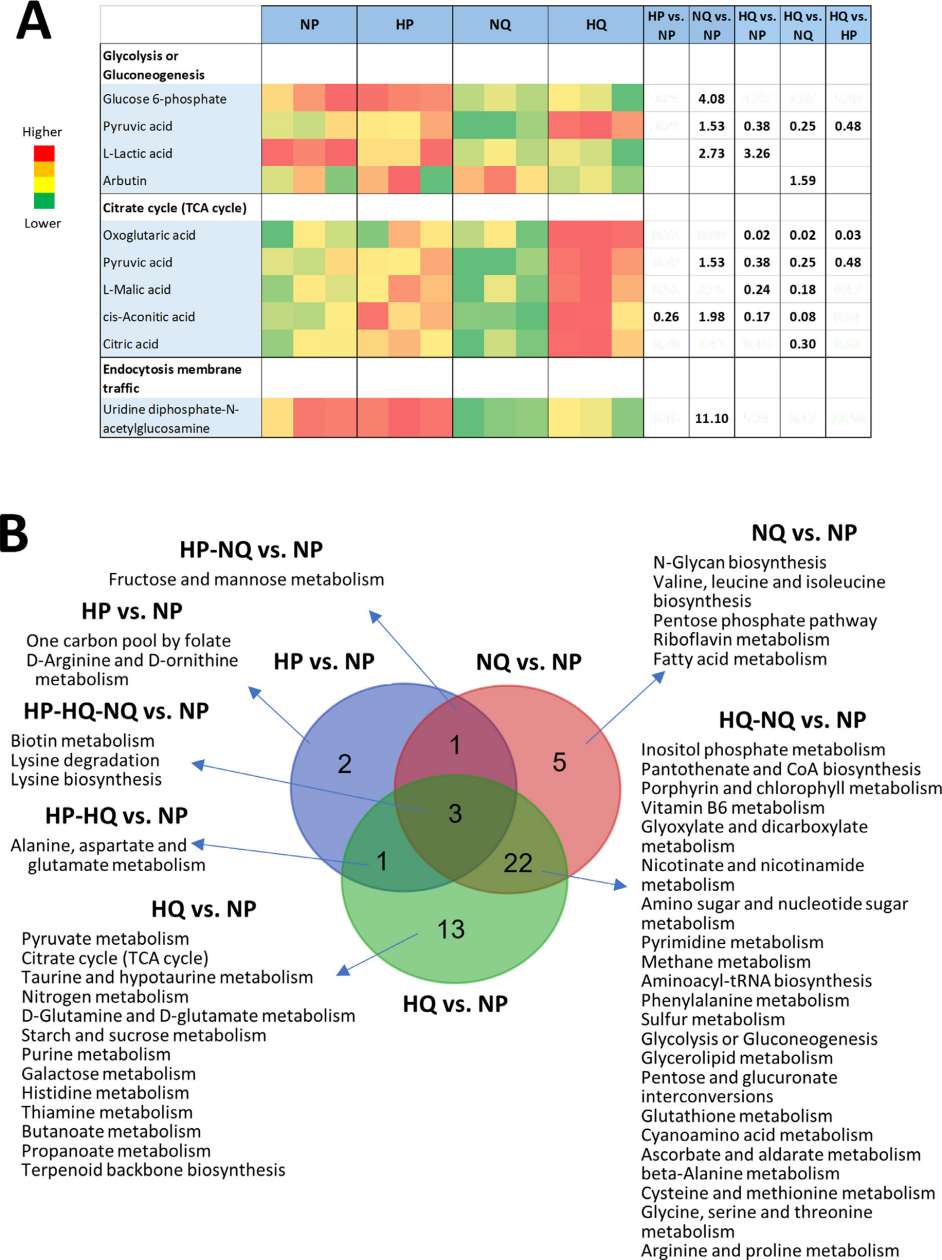
Variation in metabolite levels of proliferative HT29NIS cells in normoxic conditions (NP), proliferative HT29NIS cells in hypoxic conditions (HP), quiescent HT29NIS cells in normoxic conditions (QP) and quiescent HT29NIS cells in hypoxic conditions (HQ). (A) Heat map of the metabolite levels of significantly changed metabolites related to glycolysis, citrate cycle pathways and membrane traffic. Green indicates higher levels and red indicates lower levels. Ratios are shown for p<0.05. (B) Venn diagrams of significantly altered pathways compared to HQ are shown. A value of p <0.05 was considered significant.

Metabolomics studies were undertaken to gain further insight into the molecular mechanisms underlying NIS retention induced by quiescence and hypoxia. Data variations in metabolite levels and related pathways are shown in Supplementary Data 2. Supplementary Fig. 8 summarizes significant metabolite variations. In Fig. 6(A), the heatmap indicates variations of the metabolites related to glycolysis and TCA-cycle pathways. Our results show that pyruvic acid is increased in quiescent cells under normoxic conditions and decreased in quiescent cells under hypoxic conditions. Lactic acid is increased in quiescent cells in hypoxic and normoxic conditions. Most metabolites of the TCA-cycle are decreased in quiescent cells under hypoxic conditions. All the pathway analysis results are summarized in Fig. 6 using Venn diagrams. Fig. 6(B) shows the modified pathways in the different conditions compared to proliferative cells in normoxic conditions. Supplementary Fig. 9 compares quiescent cells in hypoxic conditions to all other conditions). This analysis suggests that quiescence induces significantly more changes in the metabolic pathways than hypoxia. Hypoxia induces increases of certain amino acid levels (for example, arginine and lysine) in proliferative cells and decreases of others (aspartate and glutamate) in quiescent cells. Our metabolomics experiments provide preliminary results regarding the changes in cellular metabolism that are induced by our experimental conditions.

## Discussion

The applicability of NIS gene therapy for tumors of extrathyroidal origin has been hampered by the heterogenous NIS expression within tumors resulting in a reduced radioiodide uptake ability and treatment efficacy. In the present study, we addressed whether the heterogeneous expression and uptake of NIS could be correlated to the heterogeneous characteristics of tumors in terms of hypoxia and quiescence, two key features of the tumor microenvironment. By developing several xenograft models with exogenous NIS-expressing-cancer cells, we showed that NIS-mediated radioiodide uptake and NIS localization at the plasma membrane of cancer cells are impaired in intratumoral areas exhibiting hypoperfusion, a well-known inducer of hypoxia and quiescence. In line with this observation, a lower uptake ability of the xenograft was observed in areas of the tumor presenting a lower proliferative (Ki-67) index as well as a higher HIF-1 alpha staining for hypoxia. Given the complexity in analyzing the separate and combined influence of hypoxia and quiescence on NIS activity and proper localization at the plasma membrane using in vivo models, we then performed in vitro studies on the HT29NIS cancer cell line. The effects of hypoxia and quiescence, separately and in combination, on NIS uptake ability, subcellular localization, and expression were therefore assessed in NIS-expressing cancer cells. In addition, to get a better understanding of the underlying molecular mechanisms through which hypoxia and quiescence may impair NIS function, we also undertook untargeted proteomics and metabolomics analyses. Altogether, our data provide evidence that both hypoxia and quiescence impair NIS-mediated uptake and NIS expression at the plasma membrane of cancer cells. More precisely, NIS activity is inhibited by both factors to a different extent and through distinct patterns of mis-localization of the protein to intracellular compartments. Proteomics and metabolomics experiments provided preliminary molecular features that are related to post-translational NIS regulation induced by hypoxia and/or quiescence.

**Fig. 7:**
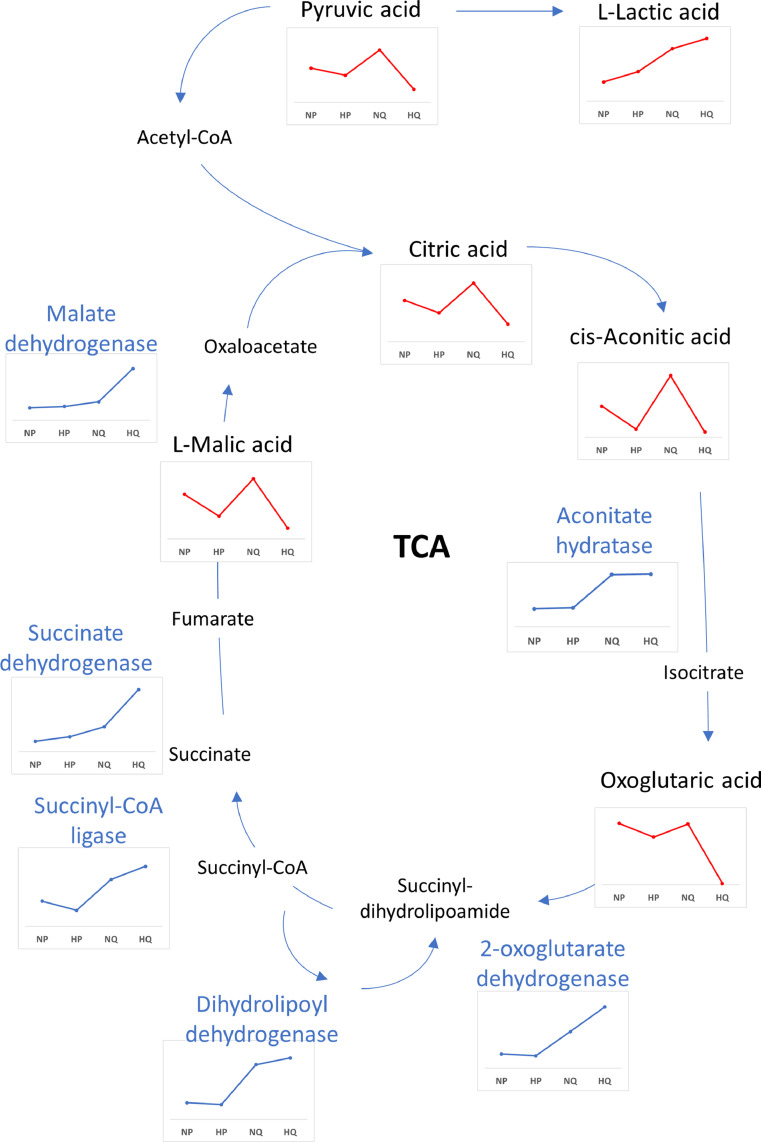
Regulation of the TriCarboxylic Acid (TCA) Cycle in proliferative HT29NIS cells in normoxic conditions (NP), proliferative HT29NIS cells in hypoxic conditions (HP), quiescent HT29NIS cells in normoxic conditions (QP) and quiescent HT29NIS cells in hypoxic conditions (HQ). Schematic representation with the expression profiles of metabolites and enzymes detected.

Quiescence induced many changes in the HT29NIS cell proteome, in particular and as expected, a reduction in the levels of proteins that are related to cell cycle control of chromosomal replication. Modifications were also observed in additional pathways (cellular metabolism, signaling and trafficking). By contrast, hypoxia induced few global proteome variations. Similar global changes of metabolic pathways were found between quiescence and hypoxia, which is consistent with the observation that hypoxia is as a modulator of cell proliferation [61]. In addition, a focus on the TCA-cycle (Fig. 7) showed that related enzymes were mainly induced by quiescence. Slightly reduced levels in TCA-related metabolites and pyruvic acid were found in proliferative cells under hypoxic conditions when compared to normoxic conditions, as expected. Such metabolites exhibited increased levels in quiescent cells in normoxic conditions when compared to proliferative cells, in correlation with higher levels of the corresponding enzymes. Finally, TCA-related metabolites and pyruvic acid levels were strongly decreased in quiescent cells under hypoxic conditions. The extent of this effect reveals more than cumulative changes and could explain the decrease in the cell viability. To our knowledge, no other “omic” study has been performed using similar experimental conditions. Downregulation of the temporal profiles of TCA-cycle enzymes in the transition from quiescence to proliferation in response to IL-3 was previously reported by Lee and collaborators [62]. Although these results differ from ours, the differences in both the experimental conditions as well as the cell lines tested (non-malignant murine pro-B lymphocyte cell line), may account for this apparent discrepancy.

In the clinic, the targeted radioactive iodine therapy of extrathyroidal tumors using hNIS as a therapeutic gene has been performed through NIS transfer using an adenovirus vector in which NIS expression was driven by the cytomegalovirus (CMV) promoter. To gain better insight into the mechanisms controlling NIS activity and subcellular localization within non-thyroidal tumor cells, we established cancer cells in which NIS expression is under the control of the constitutive CMV promoter [10]. Several lines of evidence from the literature suggest that CMV promoter activity may vary according to different factors including components from the tumor microenvironment. Using a human anaplastic thyroid carcinoma cell line transfected with a plasmid encoding hNIS, Kim and collaborators linked CMV promoter activation in response to doxorubicin to that of NF-kB [63]. However, controversies exist surrounding the link to hypoxia and quiescence. Both of these tumor microenvironment components were reported by D'Ignazio to induce NF-κB activation [64], whereas reduced expression of proteins under the CMV promoter was described to occur only in response to hypoxia by the Wendland group [65]. In our study, western-blot analyses indicated that NIS expression is reduced by hypoxia but remains unaffected by quiescence. Although no variation in NIS-encoding RNA is expected in response to quiescence, lower NIS-encoding RNA levels and other post-translational mechanisms may account for the decreased NIS-protein level induced by hypoxia.

Parameters other than the CMV promoter activity may account for the reduced expression of NIS at the plasma membrane of cancer cells. The intracellular expression of NIS has already been reported in tumor cells of different origins including breast cancer and thyroid [66], [67], [68], [69], [70]. Although the strong intracellular staining observed in breast and thyroid cancers proved to be mainly due to nonspecific binding of the antibodies by our group [71], we cannot rule out that altered NIS localization in thyroid cancer cells could contribute to lower its expression at the plasma membrane and to reduce the NIS-mediated iodide uptake. NIS localization at the plasma membrane has been reported to be tightly regulated [6] and several sorting motifs have been proposed [6, 32], but the underlying molecular mechanisms remain poorly understood.

Studies on different membrane proteins have provided evidence that hypoxia modulates endocytosis, integrins and Na,K-ATPase [72]. In alveolar epithelial cells, hypoxia-induced endocytosis of Na,K-ATPase was shown to be mediated by mitochondrial reactive oxygen species, PKC-zeta [73] and RhoA activation [74], suggesting that phosphorylation plays a role in Na,K-ATPase endocytosis. Phosphorylation sites have also been identified on the NIS protein [75]. In addition, Kiang and collaborators reported that hypoxia activates the phosphorylating enzyme PKC [76]. Phosphorylation sites of NIS could therefore be involved in hypoxia-induced NIS mislocalization. Although none of the five phosphorylation sites of NIS studied by Vadysirisack appeared to be involved in the regulation of the protein at the plasma membrane [75], others may be involved and remain to be studied.

Glycosylation-dependent mechanisms have also been involved in the membrane localization of hNIS and in its uptake activity [77]. Recently, a particular cellular metabolite (uridine diphosphate N-acetylglucosamine ) was even shown to directly control endocytic traffic proteins by regulating glycosylation [78], Of interest, we found levels of this metabolite to be markedly increased in quiescent cells, suggesting that quiescence could induce NIS endocytosis and reduce tumor cell uptake ability.

Along this line, our proteomics analyses indicate a marked effect of quiescence combined with hypoxia on the cellular trafficking pathways (i.e. Caveolar-mediated endocytosis signaling or phagosome maturation). Interestingly, similar inhibitions in trafficking pathways (in particular Caveolar-mediated endocytosis and clathrin-mediated endocytosis signaling) were previously reported by our group in thyroids where the uptake ability was inhibited with either NaI or iodinated contrast agents [79]. It is therefore tempting to speculate on the existence of common mechanisms controlling NIS localization and activity/inhibition between thyroid and NIS-expressing extrathyroidal cancer cells.

Beyond cellular trafficking, the tumor microenvironment may affect protein degradation. Recent evidence demonstrates that autophagy is associated with NIS expression at the plasma membrane in thyroid cancers [80] and that HMGB1-mediated autophagy could regulate NIS protein degradation [81]. Considering the cross talk between hypoxia and autophagy mediated by HIFα and NF-κB [82], our results suggest that such a mechanism could also be involved in reducing NIS-expression at the plasma membrane in cells under hypoxic conditions. However, because autophagy is a dynamic process with autophagosomes constantly forming and disappearing, analyzing the levels of key autophagic factors such as LC3 through proteomics was not appropriate to quantify this phenomenon. Dedicated experiments will therefore be required to measure the autophagic flux and assess its potential involvement in NIS regulation

Although informative, our proteomic analyses failed to detect NIS proteins and its post-translational modifications. Several key protein modifications (phosphorylation, glycosylation, ubiquitination of tyrosine residues, etc.) have been described by different groups, including our own, to critically affect NIS interactions with intracellular partners, its subsequent proper localization on the plasma membrane, as well as its activity [32, 75, 83]. Although beyond the scope of the present work, a study simultaneously addressing the transcriptional, translational, and post-translational levels of NIS regulation in response to hypoxia and quiescence could be very informative.

In poorly differentiated thyroid carcinoma, very low iodide uptakes have been reported. Despite very low NIS-encoding RNA[84], the NIS-posttranscriptional regulation by hypoxia and quiescence that occur in thyroid tumor cells cannot be excluded as a contributing factor to the complete lack of iodide uptake. In well-differentiated thyroid carcinomas (papillary carcinoma or follicular carcinoma), variable reductions in NIS-encoding RNA levels have been reported [84]. According to metabolomics studies on thyroid cancers, tumor cells have increased glycolytic activity and decreased TCA cycle products that are related to the Warburg effect and hypoxia [85]. We postulate that NIS targeting at the plasma membrane could be impaired in well-differentiated thyroid tumors by molecular mechanisms similar to those described in this study. Thus, the tumor microenvironment could promote lower NIS-mediated iodide uptake in well-differentiated thyroid tumors and metastases.Improving our understanding of the fine regulation of NIS may have consequences of paramount importance for cancers with low expression of membranous NIS, in order to improve radioiodine therapy.

One limitation of NIS-mediated gene therapy is that, in most preclinical studies, efficient radiotherapy requires radioactive doses proportionally higher than those currently used in humans and considered to be safe. For most reported preclinical studies, xenografts derived from tumor cell lines were used. Most of these cellular models grow quickly and xenografts are likely to be similar to those described in this study using HT29NIS cells. Quiescence and hypoxia could also lead to lower exogenous NIS expression in such xenografts. As expected from the rapid growth rate of these tumor cells lines, NIS expression in xenografts could strongly vary in the different areas of the xenograft. Using SPECT imaging, we observed similar iodide uptake limited to the border of xenografts using NIS-expressing cell lines other than HT29NIS. We can also speculate that quiescence and hypoxia are more prominent in such xenografts than in most human neoplasms and may therefore contribute to underestimate the antitumor effects using tumor cell line-based preclinical models in NIS gene radiotherapy studies.

In conclusion, our study provides the first line of evidence that hypoxia and quiescence strongly impair NIS-mediated uptake by post-transcriptional mechanisms that mainly alter NIS-protein expression at the plasma membrane. The tumor microenvironment is complex and additional studies exploring the link between microenvironment factors, signal transduction and metabolic pathways may help elucidate the pathological mechanisms involved in NIS-mediated therapy. Our observations may have implications for the clinical management of patients affected by differentiated thyroid carcinomas and non-thyroidal cancers, where controlling environmental factors may potentiate the efficacy of radioiodine therapy.

## CRediT authorship contribution statement

**Fabio Castillo-Rivera:** Methodology, Investigation, Validation, Writing - original draft. **Alejandro Ondo-Méndez:** Conceptualization, Methodology, Investigation, Validation, Formal analysis, Writing - original draft, Supervision, Funding acquisition. **Julien Guglielmi:** Methodology, Investigation, Validation. **Jean-Marie Guigonis:** Methodology, Investigation, Validation, Formal analysis. **Lun Jing:** Methodology, Investigation, Validation, Formal analysis. **Sabine Lindenthal:** Methodology, Investigation, Validation, Formal analysis. **Andrea Gonzalez:** Validation, Formal analysis. **Diana López:** Validation, Formal analysis. **Béatrice Cambien:** Methodology, Formal analysis, Writing - review & editing. **Thierry Pourcher:** Conceptualization, Methodology, Formal analysis, Writing - review & editing, Supervision, Resources, Project administration, Funding acquisition.

## Appendix Supplementary materials

Image, application 1

Image, application 2

Image, application 3

Image, application 4

Image, application 5

## References

[1] Ravera S., Reyna-Neyra A., Ferrandino G., Amzel L.M., Carrasco N. (2017). The sodium/iodide symporter (NIS): molecular physiology and preclinical and clinical applications. Annu. Rev. Physiol..

[2] Darrouzet E., Lindenthal S., Marcellin D., Pellequer J.L., Pourcher T. (2014). The sodium/iodide symporter: state of the art of its molecular characterization. Biochim. Biophys. Acta.

[3] Portulano C., Paroder-Belenitsky M., Carrasco N. (2014). The Na+/I- symporter (NIS): mechanism and medical impact. Endocr. Rev..

[4] Haugen B.R., Alexander E.K., Bible K.C., Doherty G.M., Mandel S.J., Nikiforov Y.E., Pacini F., Randolph G.W., Sawka A.M., Schlumberger M., Schuff K.G., Sherman S.I., Sosa J.A., Steward D.L., Tuttle R.M., Wartofsky L. (2016). 2015 American thyroid association management guidelines for adult patients with thyroid nodules and differentiated thyroid cancer: the American thyroid association guidelines task force on thyroid nodules and differentiated thyroid cancer. Thyroid.

[5] Choudhury P.S., Gupta M. (2018). Differentiated thyroid cancer theranostics: radioiodine and beyond. Br. J. Radiol..

[6] Martin M., Geysels R.C., Peyret V., Bernal Barquero C.E., Masini-Repiso A.M., Nicola J.P. (2019). Implications of Na(+)/I(-) symporter transport to the plasma membrane for thyroid hormonogenesis and radioiodide therapy. J. Endocr. Soc..

[7] Lakshmanan A., Scarberry D., Shen D.H., Jhiang S.M. (2014). Modulation of sodium iodide symporter in thyroid cancer. Horm. Cancer.

[8] Reiners C., Hanscheid H., Luster M., Lassmann M., Verburg F.A. (2011). Radioiodine for remnant ablation and therapy of metastatic disease. Nat. Rev. Endocrinol..

[9] Dai G., Levy O., Carrasco N. (1996). Cloning and characterization of the thyroid iodide transporter. Nature.

[10] Perron B., Rodriguez A.M., Leblanc G., Pourcher T. (2001). Cloning of the mouse Sodium Iodide Symporter (mNIS) and its expression in the mammary gland and other tissues. J. Endocrinol..

[11] Smanik P.A., Liu Q., Furminger T.L., Ryu K., Xing S., Mazzaferri E.L., Jhiang S.M. (1996). Cloning of the human sodium lodide symporter. Biochem. Biophys. Res. Commun..

[12] Dingli D., Russell S.J., Morris J.C. (2003). In vivo imaging and tumor therapy with the sodium iodide symporter. J. Cell Biochem..

[13] Ahn B.C. (2012). Sodium iodide symporter for nuclear molecular imaging and gene therapy: from bedside to bench and back. Theranostics.

[14] Penheiter A.R., Russell S.J., Carlson S.K. (2012). The sodium iodide symporter (NIS) as an imaging reporter for gene, viral, and cell-based therapies. Curr. Gene Ther..

[15] Dingli D., Diaz R.M., Bergert E.R., O'Connor M.K., Morris J.C., Russell S.J. (2003). Genetically targeted radiotherapy for multiple myeloma. Blood.

[16] Dingli D., Peng K.W., Harvey M.E., Greipp P.R., O'Connor M.K., Cattaneo R., Morris J.C., Russell S.J. (2004). Image-guided radiovirotherapy for multiple myeloma using a recombinant measles virus expressing the thyroidal sodium iodide symporter. Blood.

[17] Faivre J., Clerc J., Gerolami R., Herve J., Longuet M., Liu B., Roux J., Moal F., Perricaudet M., Brechot C. (2004). Long-term radioiodine retention and regression of liver cancer after sodium iodide symporter gene transfer in wistar rats. Cancer Res..

[18] Mandell R.B., Mandell L.Z., Link C.J. (1999). Radioisotope concentrator gene therapy using the sodium/iodide symporter gene. Cancer Res..

[19] Boland A., Ricard M., Opolon P., Bidart J.M., Yeh P., Filetti S., Schlumberger M., Perricaudet M. (2000). Adenovirus-mediated transfer of the thyroid sodium/iodide symporter gene into tumors for a targeted radiotherapy. Cancer Res..

[20] Spitzweg C., Dietz A.B., O'Connor M.K., Bergert E.R., Tindall D.J., Young C.Y., Morris J.C. (2001). In vivo sodium iodide symporter gene therapy of prostate cancer. Gene Ther..

[21] Cho J.Y., Shen D.H., Yang W., Williams B., Buckwalter T.L., La Perle K.M., Hinkle G., Pozderac R., Kloos R., Nagaraja H.N., Barth R.F., Jhiang S.M. (2002). In vivo imaging and radioiodine therapy following sodium iodide symporter gene transfer in animal model of intracerebral gliomas. Gene Ther..

[22] Peerlinck I., Merron A., Baril P., Conchon S., Martin-Duque P., Hindorf C., Burnet J., Quintanilla M., Hingorani M., Iggo R., Lemoine N.R., Harrington K., Vassaux G. (2009). Targeted radionuclide therapy using a Wnt-targeted replicating adenovirus encoding the Na/I symporter. Clin. Cancer Res..

[23] Barton K.N., Stricker H., Brown S.L., Elshaikh M., Aref I., Lu M., Pegg J., Zhang Y., Karvelis K.C., Siddiqui F., Kim J.H., Freytag S.O., Movsas B. (2008). Phase I study of noninvasive imaging of adenovirus-mediated gene expression in the human prostate. Mol. Ther..

[24] Gengenbacher N., Singhal M., Augustin H.G. (2017). Preclinical mouse solid tumour models: status quo, challenges and perspectives. Nat. Rev. Cancer.

[25] Kogai T., Endo T., Saito T., Miyazaki A., Kawaguchi A., Onaya T. (1997). Regulation by thyroid-stimulating hormone of sodium/iodide symporter gene expression and protein levels in FRTL-5 cells. Endocrinology.

[26] Riedel C., Levy O., Carrasco N. (2001). Post-transcriptional regulation of the sodium/iodide symporter (NIS) by thyrotropin. J. Biol. Chem..

[27] Eng P.H., Cardona G.R., Previti M.C., Chin W.W., Braverman L.E. (2001). Regulation of the sodium iodide symporter by iodide in FRTL-5 cells. Eur. J. Endocrinol..

[28] Knostman K.A., McCubrey J.A., Morrison C.D., Zhang Z., Capen C.C., Jhiang S.M. (2007). PI3K activation is associated with intracellular sodium/iodide symporter protein expression in breast cancer. BMC Cancer.

[29] Smith V.E., Read M.L., Turnell A.S., Watkins R.J., Watkinson J.C., Lewy G.D., Fong J.C., James S.R., Eggo M.C., Boelaert K., Franklyn J.A., McCabe C.J. (2009). A novel mechanism of sodium iodide symporter repression in differentiated thyroid cancer. J. Cell Sci..

[30] Zhang Z., Liu Y.Y., Jhiang S.M. (2005). Cell surface targeting accounts for the difference in iodide uptake activity between human Na+/I- symporter and rat Na+/I- symporter. J. Clin. Endocrinol. Metab..

[31] Dayem M., Basquin C., Navarro V., Carrier P., Marsault R., Chang P., Huc S., Darrouzet E., Lindenthal S., Pourcher T. (2008). Comparison of expressed human and mouse sodium/iodide symporters reveals differences in transport properties and subcellular localization. J. Endocrinol..

[32] Darrouzet E., Graslin F., Marcellin D., Tcheremisinova I., Marchetti C., Salleron L., Pognonec P., Pourcher T. (2016). A systematic evaluation of sorting motifs in the sodium-iodide symporter (NIS). Biochem J.

[33] Martin M., Modenutti C.P., Peyret V., Geysels R.C., Darrouzet E., Pourcher T., Masini-Repiso A.M., Marti M.A., Carrasco N., Nicola J.P. (2019). A carboxy-terminal monoleucine-based motif participates in the basolateral targeting of the Na+/I- symporter. Endocrinology.

[34] Le Goas M., Paquet M., Paquirissamy A., Guglielmi J., Compin C., Thariat J., Vassaux G., Geertsen V., Humbert O., Renault J.P., Carrot G., Pourcher T., Cambien B. (2019). Improving (131)I radioiodine therapy by hybrid polymer-grafted gold nanoparticles. Int. J. Nanomed..

[35] Huang M., Batra R.K., Kogai T., Lin Y.Q., Hershman J.M., Lichtenstein A., Sharma S., Zhu L.X., Brent G.A., Dubinett S.M. (2001). Ectopic expression of the thyroperoxidase gene augments radioiodide uptake and retention mediated by the sodium iodide symporter in non-small cell lung cancer. Cancer Gene Ther..

[36] Boland A., Magnon C., Filetti S., Bidart J.M., Schlumberger M., Yeh P., Perricaudet M. (2002). Transposition of the thyroid iodide uptake and organification system in nonthyroid tumor cells by adenoviral vector-mediated gene transfers. Thyroid.

[37] Li W., Tan J., Wang P., Wu P. (2011). Cotransfected sodium iodide symporter and human tyroperoxidase genes following human telomerase reverse transcriptase promoter for targeted radioiodine therapy of malignant glioma cells. Cancer Biother. Radiopharm..

[38] Petrich T., Quintanilla-Martinez L., Korkmaz Z., Samson E., Helmeke H.J., Meyer G.J., Knapp W.H., Potter E. (2006). Effective cancer therapy with the alpha-particle emitter [211At]astatine in a mouse model of genetically modified sodium/iodide symporter-expressing tumors. Clin. Cancer Res..

[39] Willhauck M.J., Samani B.R., Wolf I., Senekowitsch-Schmidtke R., Stark H.J., Meyer G.J., Knapp W.H., Goke B., Morris J.C., Spitzweg C. (2008). The potential of 211Astatine for NIS-mediated radionuclide therapy in prostate cancer. Eur. J. Nucl. Med. Mol. Imaging.

[40] Watabe T., Kaneda-Nakashima K., Liu Y., Shirakami Y., Ooe K., Toyoshima A., Shimosegawa E., Fukuda M., Shinohara A., Hatazawa J. (2019). Enhancement of (211)At uptake via the sodium iodide symporter by the addition of ascorbic acid in targeted alpha-therapy of thyroid cancer. J. Nucl. Med..

[41] Guo R., Zhang M., Xi Y., Ma Y., Liang S., Shi S., Miao Y., Li B. (2014). Theranostic studies of human sodium iodide symporter imaging and therapy using 188Re: a human glioma study in mice. PLoS One.

[42] Zhang M., Shi S., Guo R., Miao Y., Li B. (2016). Use of rhenium-188 for in vivo imaging and treatment of human cervical cancer cells transfected with lentivirus expressing sodium iodide symporter. Oncology Rep..

[43] Guo R., Xi Y., Zhang M., Miao Y., Zhang M., Li B. (2018). Human sodium iodide transporter gene-mediated imaging and therapy of mouse glioma, comparison between (188)Re and (131)I. Oncol. Lett..

[44] Magnon C., Opolon P., Ricard M., Connault E., Ardouin P., Galaup A., Metivier D., Bidart J.M., Germain S., Perricaudet M., Schlumberger M. (2007). Radiation and inhibition of angiogenesis by canstatin synergize to induce HIF-1alpha-mediated tumor apoptotic switch. J. Clin. Investig..

[45] Hingorani M., White C.L., Zaidi S., Pandha H.S., Melcher A.A., Bhide S.A., Nutting C.M., Syrigos K.N., Vile R.G., Vassaux G., Harrington K.J. (2010). Therapeutic effect of sodium iodide symporter gene therapy combined with external beam radiotherapy and targeted drugs that inhibit DNA repair. Mol. Ther..

[46] Son S.H., Gangadaran P., Ahn B.C. (2019). A novel strategy of transferring NIS protein to cells using extracellular vesicles leads to increase in iodine uptake and cytotoxicity. Int. J. Nanomed..

[47] Opyrchal M., Allen C., Iankov I., Aderca I., Schroeder M., Sarkaria J., Galanis E. (2012). Effective radiovirotherapy for malignant gliomas by using oncolytic measles virus strains encoding the sodium iodide symporter (MV-NIS). Hum. Gene Ther..

[48] Mansfield D.C., Kyula J.N., Rosenfelder N., Chao-Chu J., Kramer-Marek G., Khan A.A., Roulstone V., McLaughlin M., Melcher A.A., Vile R.G., Pandha H.S., Khoo V., Harrington K.J. (2016). Oncolytic vaccinia virus as a vector for therapeutic sodium iodide symporter gene therapy in prostate cancer. Gene Ther..

[49] Richard-Fiardo P., Hervouet C., Marsault R., Franken P.R., Cambien B., Guglielmi J., Warnez-Soulie J., Darcourt J., Pourcher T., Colombani T., Haudebourg T., Peuziat P., Pitard B., Vassaux G. (2015). Evaluation of tetrafunctional block copolymers as synthetic vectors for lung gene transfer. Biomaterials.

[50] Urnauer S., Schmohl K.A., Tutter M., Schug C., Schwenk N., Morys S., Ziegler S., Bartenstein P., Clevert D.A., Wagner E., Spitzweg C. (2019). Dual-targeted NIS polyplexes-a theranostic strategy toward tumors with heterogeneous receptor expression. Gene Ther..

[51] Muller A.M., Schmohl K.A., Knoop K., Schug C., Urnauer S., Hagenhoff A., Clevert D.A., Ingrisch M., Niess H., Carlsen J., Zach C., Wagner E., Bartenstein P., Nelson P.J., Spitzweg C. (2016). Hypoxia-targeted 131I therapy of hepatocellular cancer after systemic mesenchymal stem cell-mediated sodium iodide symporter gene delivery. Oncotarget.

[52] Schug C., Sievert W., Urnauer S., Muller A.M., Schmohl K.A., Wechselberger A., Schwenk N., Lauber K., Schwaiger M., Multhoff G., Wagner E., Nelson P.J., Spitzweg C. (2018). External beam radiation therapy enhances mesenchymal stem cell-mediated sodium-iodide symporter gene delivery. Hum. Gene Ther..

[53] Shi S., Zhang M., Guo R., Miao Y., Li B. (2019). Bone marrow-derived mesenchymal stem cell-mediated dual-gene therapy for glioblastoma. Hum. Gene Ther..

[54] Schug C., Urnauer S., Jaeckel C., Schmohl K.A., Tutter M., Steiger K., Schwenk N., Schwaiger M., Wagner E., Nelson P.J., Spitzweg C. (2019). TGFB1-driven mesenchymal stem cell-mediated NIS gene transfer. Endocr Relat Cancer.

[55] Richard-Fiardo P., Franken P., Lamit A., Marsault R., Guglielmi J., Cambien B., Graslin F., Lindenthal S., Darcourt J., Pourcher T., Vassaux G. (2012). Normalisation to blood activity is a requirement for the accurate quantification of Na/I symporter ectopic expression by SPECT/CT in individual subjects. PLoS ONE.

[56] Cambien B., Franken P.R., Lamit A., Mauxion T., Richard-Fiardo P., Guglielmi J., Crescence L., Mari B., Pourcher T., Darcourt J., Bardies M., Vassaux G. (2014). 99mTcO4–, auger-mediated thyroid stunning: dosimetric requirements and associated molecular events. PLoS One.

[57] Coller H.A., Sang L., Roberts J.M. (2006). A new description of cellular quiescence. PLoS Biol..

[58] Sabit H., Samy M.B., Said O.A., El-Zawahri M.M. (2016). Procaine induces epigenetic changes in HCT116 colon cancer cells. Genet. Res. Int..

[59] Jing L., Guigonis J.M., Borchiellini D., Durand M., Pourcher T., Ambrosetti D. (2019). LC-MS based metabolomic profiling for renal cell carcinoma histologic subtypes. Sci. Rep..

[60] McIntyre A., Patiar S., Wigfield S., Li J.L., Ledaki I., Turley H., Leek R., Snell C., Gatter K., Sly W.S., Vaughan-Jones R.D., Swietach P., Harris A.L. (2012). Carbonic anhydrase IX promotes tumor growth and necrosis in vivo and inhibition enhances anti-VEGF therapy. Clin. Cancer Res..

[61] Hubbi M.E., Semenza G.L. (2015). Regulation of cell proliferation by hypoxia-inducible factors. Am. J. Physiol. Cell Physiol..

[62] Lee H.J., Jedrychowski M.P., Vinayagam A., Wu N., Shyh-Chang N., Hu Y., Min-Wen C., Moore J.K., Asara J.M., Lyssiotis C.A., Perrimon N., Gygi S.P., Cantley L.C., Kirschner M.W. (2017). Proteomic and metabolomic characterization of a mammalian cellular transition from quiescence to proliferation. Cell Rep..

[63] Kim K.I., Kang J.H., Chung J.K., Lee Y.J., Jeong J.M., Lee D.S., Lee M.C. (2007). Doxorubicin enhances the expression of transgene under control of the CMV promoter in anaplastic thyroid carcinoma cells. J. Nucl. Med..

[64] D'Ignazio L., Rocha S. (2016). Hypoxia Induced NF-kappaB. Cells.

[65] Wendland K., Thielke M., Meisel A., Mergenthaler P. (2015). Intrinsic hypoxia sensitivity of the cytomegalovirus promoter. Cell Death Dis..

[66] Caillou B., Troalen F., Baudin E., Talbot M., Filetti S., Schlumberger M., Bidart J.M. (1998). Na+/I- symporter distribution in human thyroid tissues: an immunohistochemical study. J Clin. Endocrinol. Metab..

[67] Jhiang S.M., Cho J.Y., Ryu K.Y., DeYoung B.R., Smanik P.A., McGaughy V.R., Fischer A.H., Mazzaferri E.L. (1998). An immunohistochemical study of Na+/I- symporter in human thyroid tissues and salivary gland tissues. Endocrinology.

[68] Castro M.R., Bergert E.R., Beito T.G., Roche P.C., Ziesmer S.C., Jhiang S.M., Goellner J.R., Morris J.C. (1999). Monoclonal antibodies against the human sodium iodide symporter: utility for immunocytochemistry of thyroid cancer [In Process Citation]. J. Endocrinol..

[69] Tazebay U.H., Wapnir I.L., Levy O., Dohan O., Zuckier L.S., Hua Zhao Q., Fu Deng H., Amenta P.S., Fineberg S., Pestell R.G., Carrasco N. (2000). The mammary gland iodide transporter is expressed during lactation and in breast cancer. Nat. Med..

[70] Wapnir I.L., van de Rijn M., Nowels K., Amenta P.S., Walton K., Montgomery K., Greco R.S., Dohan O., Carrasco N. (2003). Immunohistochemical profile of the sodium/iodide symporter in thyroid, breast, and other carcinomas using high density tissue microarrays and conventional sections. J. Clin. Endocrinol. Metab..

[71] Peyrottes I., Navarro V., Ondo-Mendez A., Marcellin D., Bellanger L., Marsault R., Lindenthal S., Ettore F., Darcourt J., Pourcher T. (2009). Immunoanalysis indicates that the sodium iodide symporter is not overexpressed in intracellular compartments in thyroid and breast cancers. Eur. J. Endocrinol..

[72] Wang Y., Ohh M. (2010). Oxygen-mediated endocytosis in cancer. J. Cell Mol. Med..

[73] Dada L.A., Chandel N.S., Ridge K.M., Pedemonte C., Bertorello A.M., Sznajder J.I. (2003). Hypoxia-induced endocytosis of Na,K-ATPase in alveolar epithelial cells is mediated by mitochondrial reactive oxygen species and PKC-zeta. J Clin Invest.

[74] Dada L.A., Novoa E., Lecuona E., Sun H., Sznajder J.I. (2007). Role of the small GTPase RhoA in the hypoxia-induced decrease of plasma membrane Na,K-ATPase in A549 cells. J. Cell Sci..

[75] Vadysirisack D.D., Chen E.S., Zhang Z., Tsai M.D., Chang G.D., Jhiang S.M. (2007). Identification of in vivo phosphorylation sites and their functional significance in the sodium iodide symporter. J. Biol. Chem..

[76] Kiang J.G., Wang X.D., Ding X.Z., Gist I.D., Smallridge R.C. (1996). Heat shock inhibits the hypoxia-induced effects on iodide uptake and signal transduction and enhances cell survival in rat thyroid FRTL-5 cells. Thyroid.

[77] Chung T., Youn H., Yeom C.J., Kang K.W., Chung J.K. (2015). Glycosylation of sodium/iodide symporter (NIS) regulates its membrane translocation and radioiodine uptake. PLoS One.

[78] Rahmani S., Defferrari M.S., Wakarchuk W.W., Antonescu C.N. (2019). Energetic adaptations: Metabolic control of endocytic membrane traffic. Traffic.

[79] Hichri M., Vassaux G., Guigonis J.M., Juhel T., Graslin F., Guglielmi J., Pourcher T., Cambien B. (2020). Proteomic analysis of iodinated contrast agent-induced perturbation of thyroid iodide uptake. J. Clin. Med..

[80] Plantinga T.S., Tesselaar M.H., Morreau H., Corssmit E.P., Willemsen B.K., Kusters B., van Engen-van Grunsven A.C., Smit J.W., Netea-Maier R.T. (2016). Autophagy activity is associated with membranous sodium iodide symporter expression and clinical response to radioiodine therapy in non-medullary thyroid cancer. Autophagy.

[81] Chai W., Ye F., Zeng L., Li Y., Yang L. (2019). HMGB1-mediated autophagy regulates sodium/iodide symporter protein degradation in thyroid cancer cells. J. Exp. Clin. Cancer Res.: CR.

[82] Ravanan P., Srikumar I.F., Talwar P. (2017). Autophagy: The spotlight for cellular stress responses. Life Sci..

[83] Vadysirisack D.D., Venkateswaran A., Zhang Z., Jhiang S.M. (2007). MEK signaling modulates sodium iodide symporter at multiple levels and in a paradoxical manner. Endocr. Relat. Cancer.

[84] Lazar V., Bidart J.M., Caillou B., Mahe C., Lacroix L., Filetti S., Schlumberger M. (1999). Expression of the Na+/I- symporter gene in human thyroid tumors: a comparison study with other thyroid-specific genes. J. Clin. Endocrinol. Metab..

[85] Xu Y., Zheng X., Qiu Y., Jia W., Wang J., Yin S. (2015). Distinct metabolomic profiles of papillary thyroid carcinoma and benign thyroid adenoma. J. Proteome Res..

