# Supplementary material for: Tumor microenvironment affects exogenous sodium/iodide symporter expression
Source: Transl Oncol. 2020 Nov 17;14(1):100937. doi: 10.1016/j.tranon.2020.100937 (PMC7679261; doi:10.1016/j.tranon.2020.100937)
Supplement: Supplementary file 1 [file mmc1.pptx]

## Slide 1
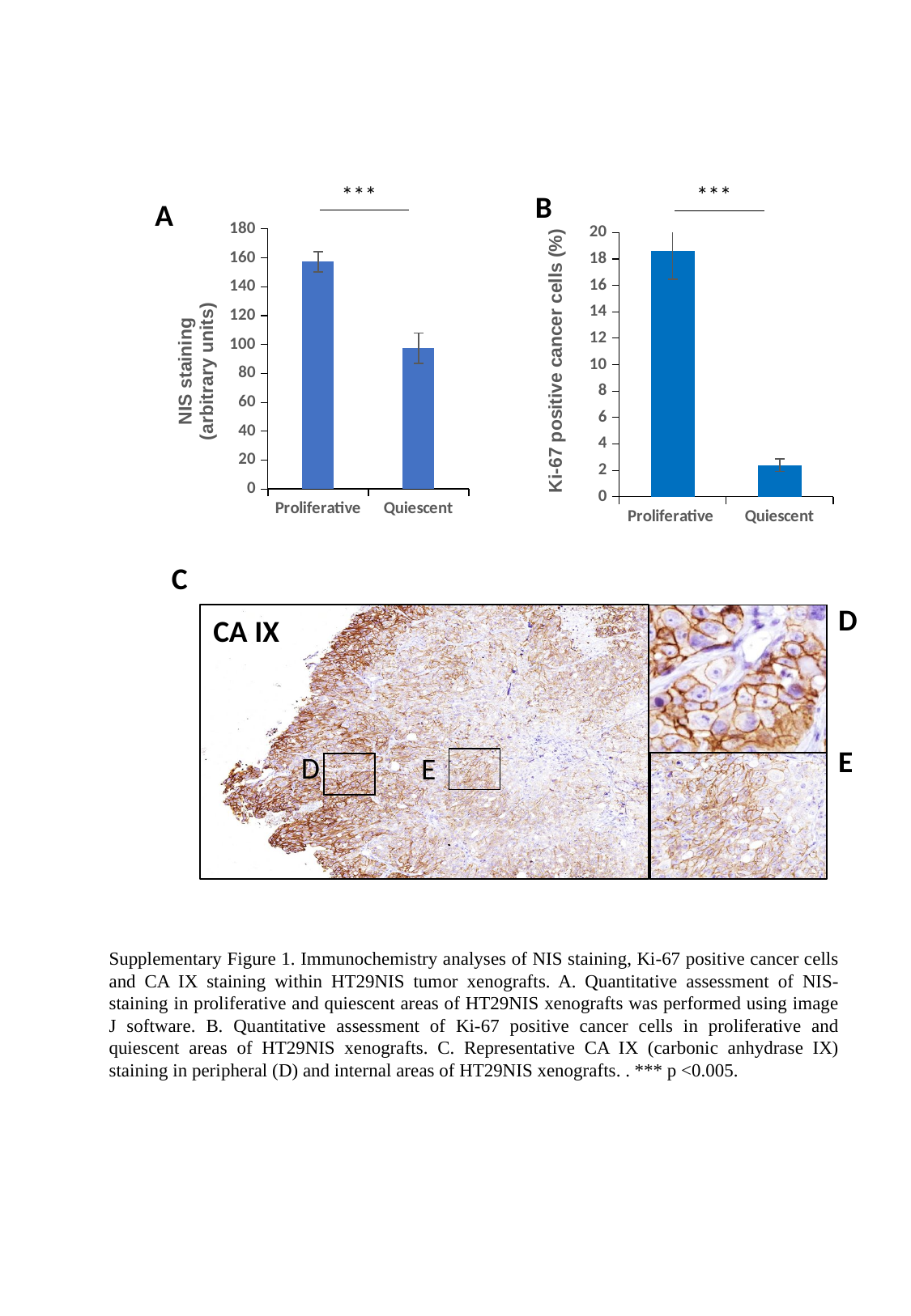

### Chart
| Category | |
|---|---|
| Proliferative | 18.61 |
| Quiescent | 2.38 |***
***
B
A
### Chart
| Category | |
|---|---|
| Proliferative | 157.2746875 |
| Quiescent | 97.46687499999999 |C
D
CA IX
E
D
E
Supplementary Figure 1. Immunochemistry analyses of NIS staining, Ki-67 positive cancer cells and CA IX staining within HT29NIS tumor xenografts. A. Quantitative assessment of NIS-staining in proliferative and quiescent areas of HT29NIS xenografts was performed using image J software. B. Quantitative assessment of Ki-67 positive cancer cells in proliferative and quiescent areas of HT29NIS xenografts. C. Representative CA IX (carbonic anhydrase IX) staining in peripheral (D) and internal areas of HT29NIS xenografts. . *** p <0.005.

## Slide 2
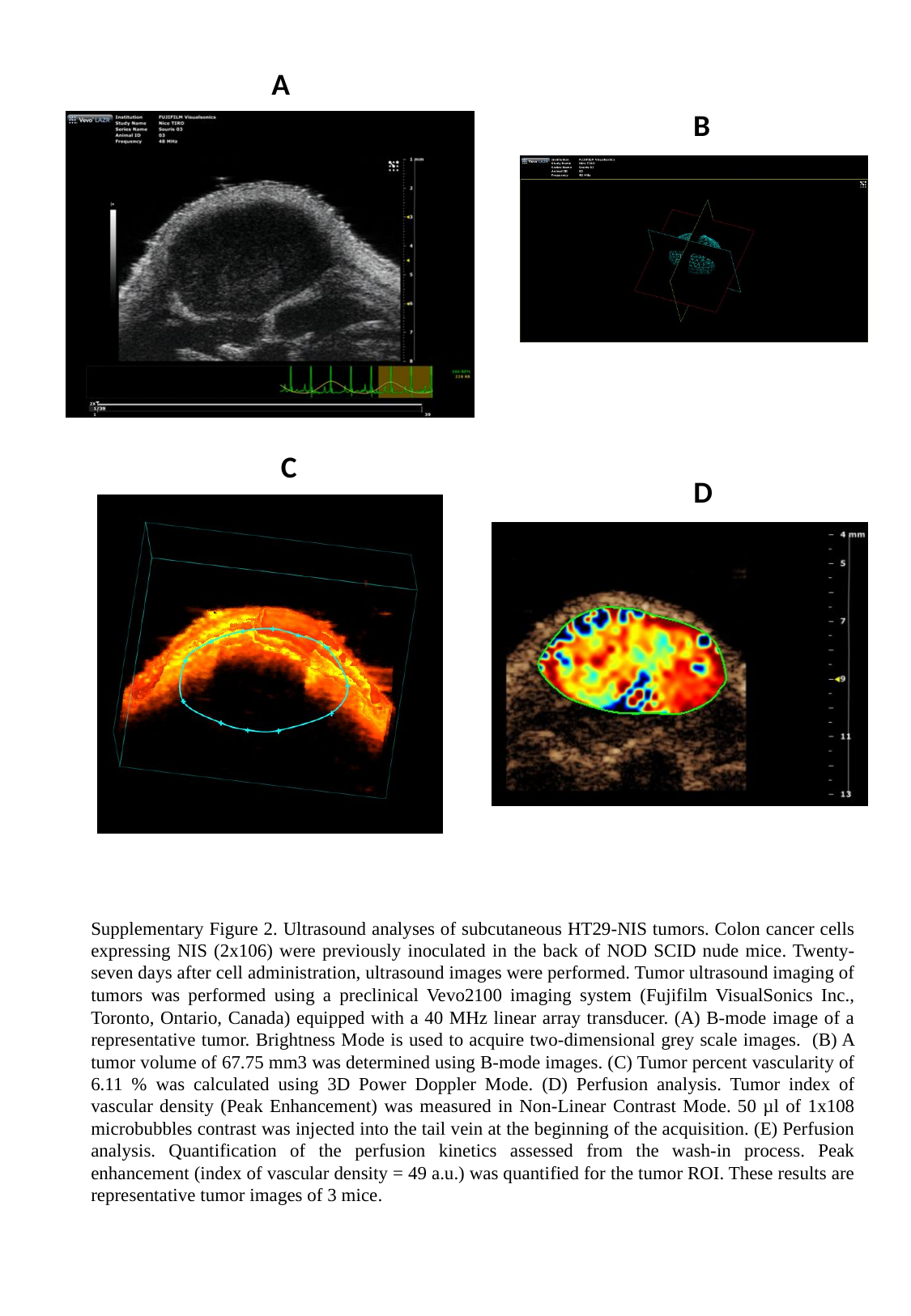

A
B
C
D
Supplementary Figure 2. Ultrasound analyses of subcutaneous HT29-NIS tumors. Colon cancer cells expressing NIS (2x10^6^) were previously inoculated in the back of NOD SCID nude mice. Twenty-seven days after cell administration, ultrasound images were performed. Tumor ultrasound imaging of tumors was performed using a preclinical Vevo2100 imaging system (Fujifilm VisualSonics Inc., Toronto, Ontario, Canada) equipped with a 40 MHz linear array transducer. (A) B-mode image of a representative tumor. Brightness Mode is used to acquire two-dimensional grey scale images. (B) A tumor volume of 67.75 mm3 was determined using B-mode images. (C) Tumor percent vascularity of 6.11 % was calculated using 3D Power Doppler Mode. (D) Perfusion analysis. Tumor index of vascular density (Peak Enhancement) was measured in Non-Linear Contrast Mode. 50 µl of 1x10^8^ microbubbles contrast was injected into the tail vein at the beginning of the acquisition. (E) Perfusion analysis. Quantification of the perfusion kinetics assessed from the wash-in process. Peak enhancement (index of vascular density = 49 a.u.) was quantified for the tumor ROI. These results are representative tumor images of 3 mice.

## Slide 3
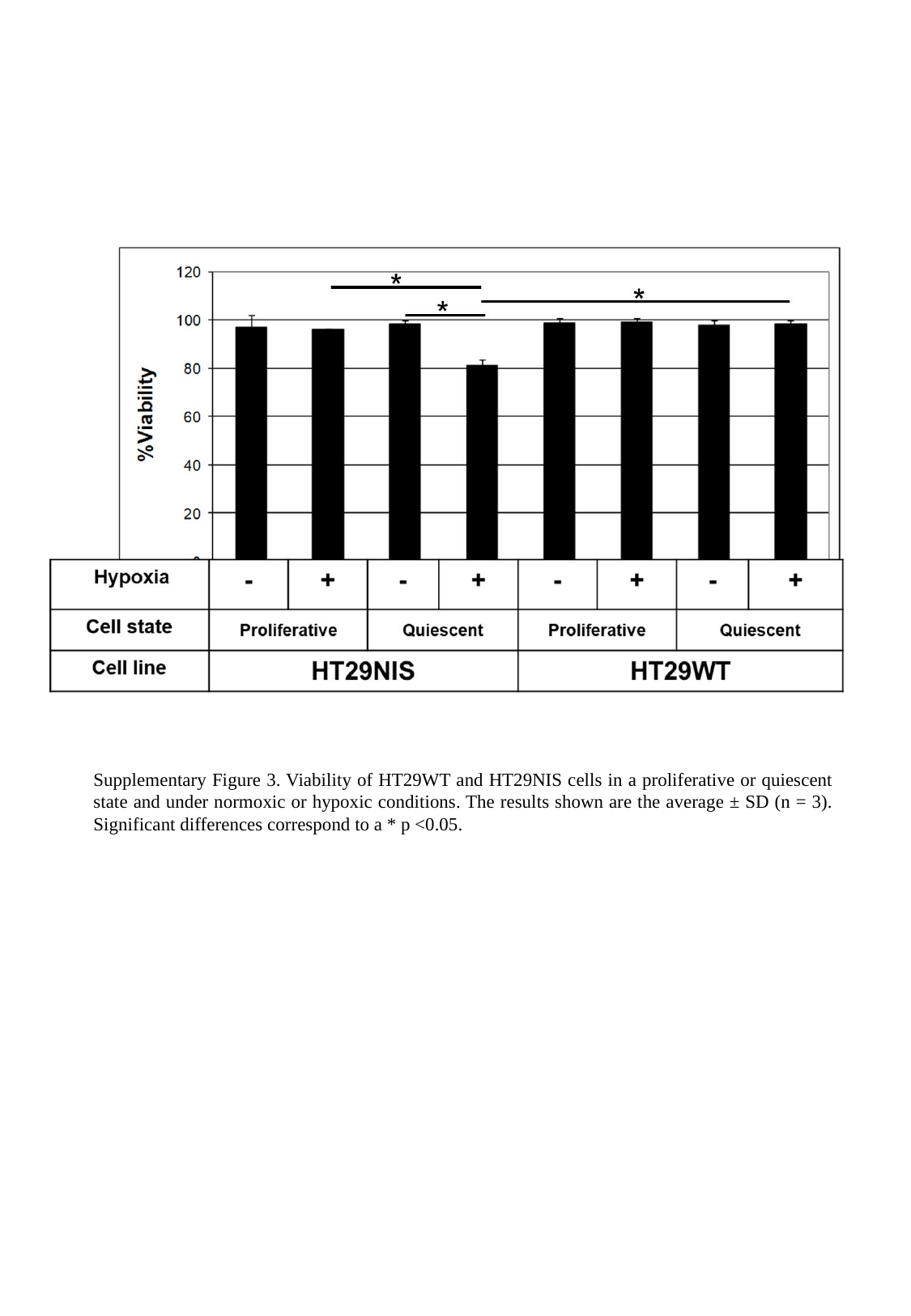

Supplementary Figure 3. Viability of HT29WT and HT29NIS cells in a proliferative or quiescent state and under normoxic or hypoxic conditions. The results shown are the average ± SD (n = 3). Significant differences correspond to a * p <0.05.

## Slide 4
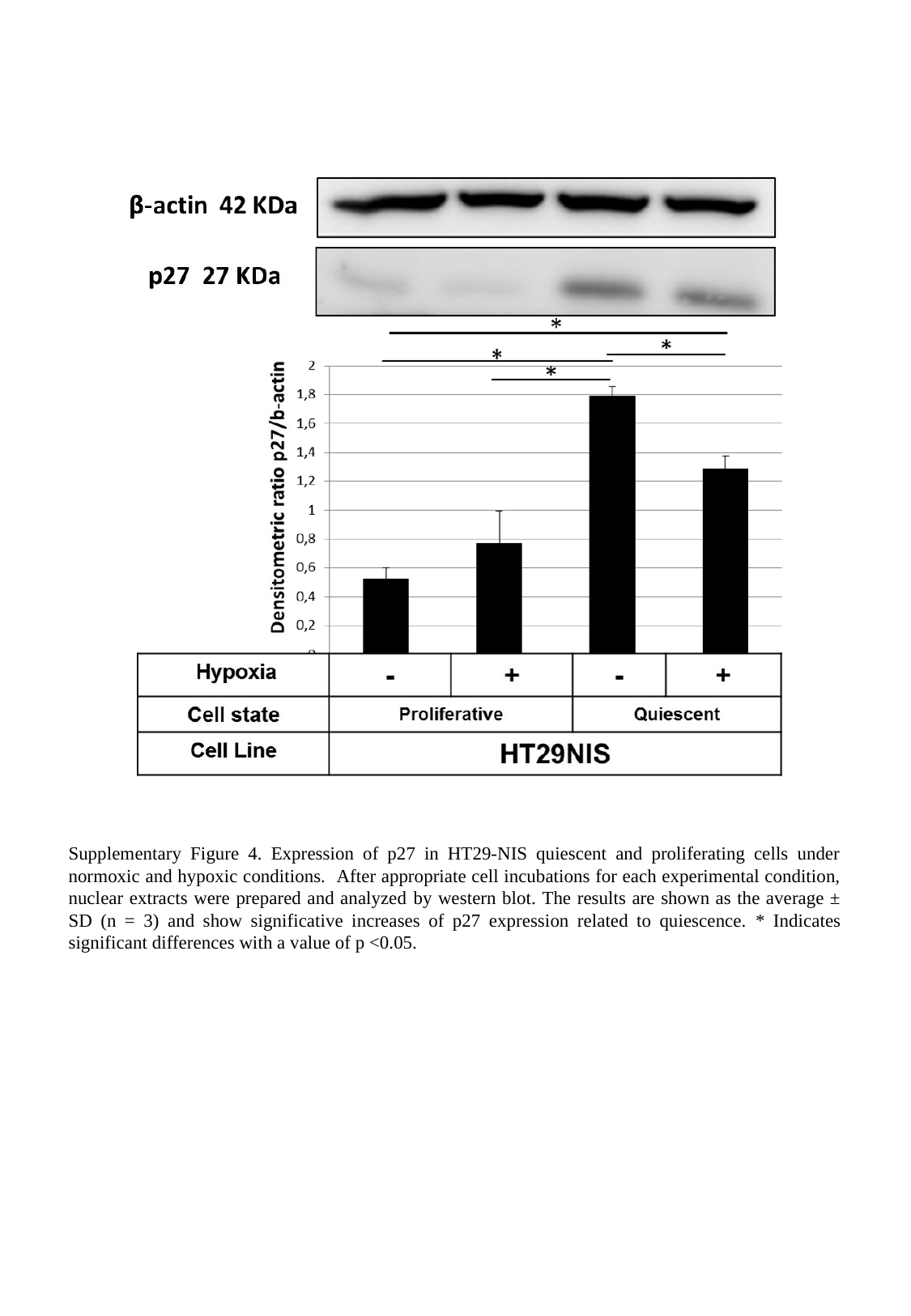

Supplementary Figure 4. Expression of p27 in HT29-NIS quiescent and proliferating cells under normoxic and hypoxic conditions. After appropriate cell incubations for each experimental condition, nuclear extracts were prepared and analyzed by western blot. The results are shown as the average ± SD (n = 3) and show significative increases of p27 expression related to quiescence. * Indicates significant differences with a value of p <0.05.

## Slide 5
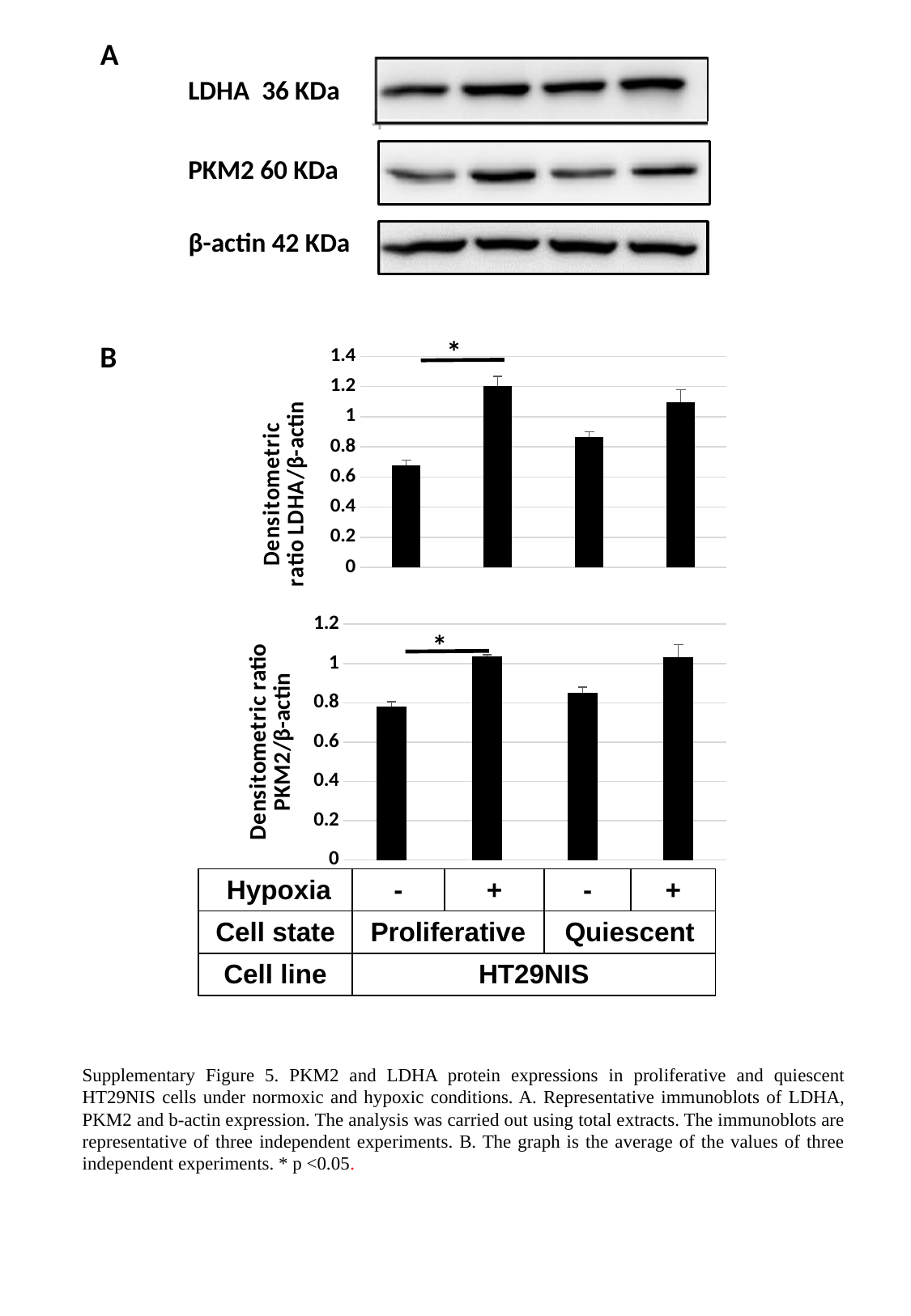

A
LDHA 36 KDa
PKM2 60 KDa
β-actin 42 KDa
*
### Chart
| Category | |
|---|---|
| NIS-NP | 0.6787912317035655 |
| NIS-HP | 1.20323924754073 |
| NIS-QN | 0.8627093268439164 |
| NIS-QH | 1.0978779764456776 |
### Chart
| Category | |
|---|---|
| NIS-NP | 0.7806408535849014 |
| NIS-HP | 1.0374919704814216 |
| NIS-QN | 0.8527199999999999 |
| NIS-QH | 1.0325968988093397 |*
B
| Hypoxia | - | + | - | + |
| --- | --- | --- | --- | --- |
| Cell state | Proliferative | | Quiescent | |
| Cell line | HT29NIS | | | |
Supplementary Figure 5. PKM2 and LDHA protein expressions in proliferative and quiescent HT29NIS cells under normoxic and hypoxic conditions. A. Representative immunoblots of LDHA, PKM2 and b-actin expression. The analysis was carried out using total extracts. The immunoblots are representative of three independent experiments. B. The graph is the average of the values of three independent experiments. * p <0.05.

## Slide 6
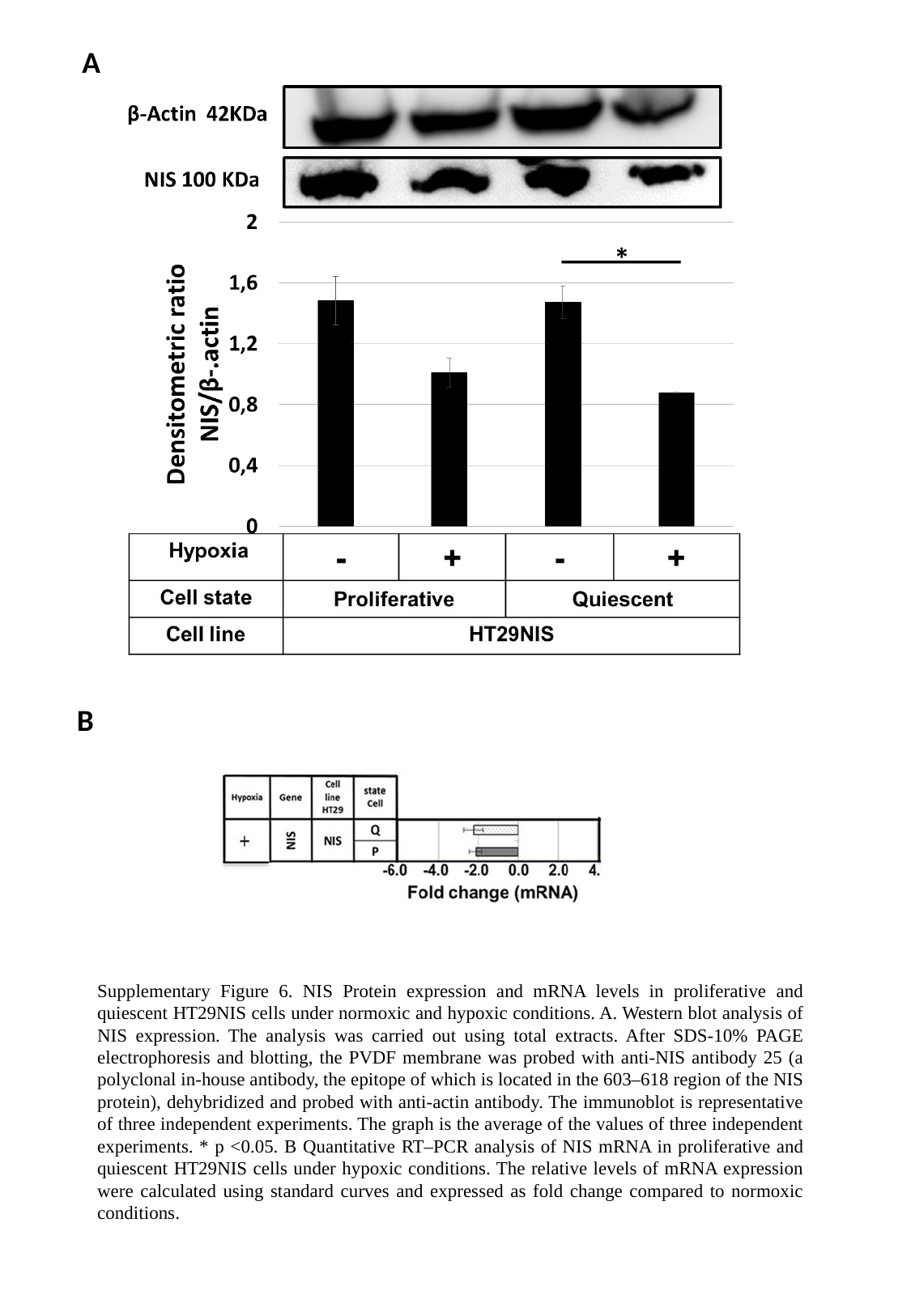

A
B
Supplementary Figure 6. NIS Protein expression and mRNA levels in proliferative and quiescent HT29NIS cells under normoxic and hypoxic conditions. A. Western blot analysis of NIS expression. The analysis was carried out using total extracts. After SDS-10% PAGE electrophoresis and blotting, the PVDF membrane was probed with anti-NIS antibody 25 (a polyclonal in-house antibody, the epitope of which is located in the 603–618 region of the NIS protein), dehybridized and probed with anti-actin antibody. The immunoblot is representative of three independent experiments. The graph is the average of the values of three independent experiments. * p <0.05. B Quantitative RT–PCR analysis of NIS mRNA in proliferative and quiescent HT29NIS cells under hypoxic conditions. The relative levels of mRNA expression were calculated using standard curves and expressed as fold change compared to normoxic conditions.

## Slide 7
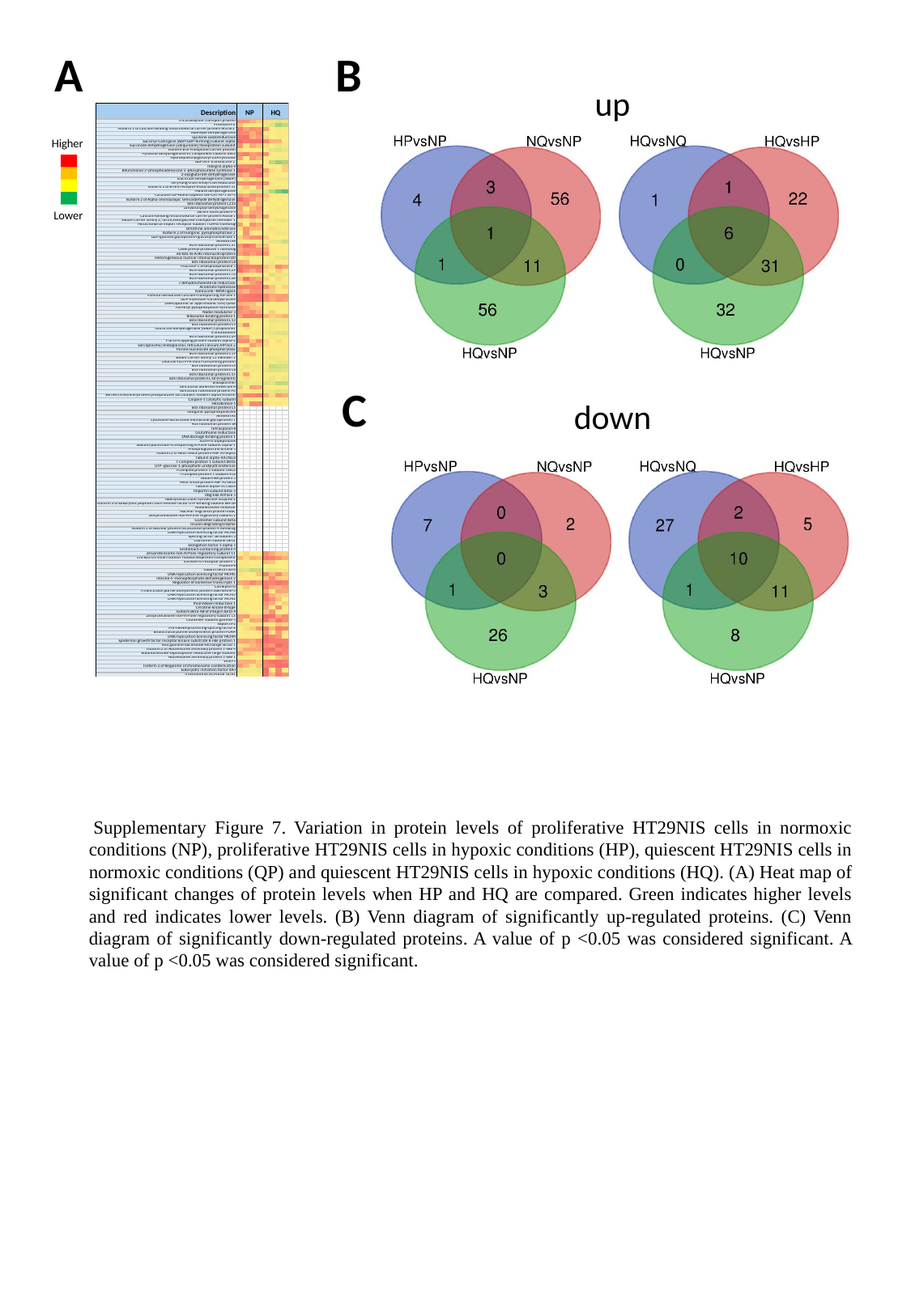

A
B
up
Higher
Lower
C
down
 Supplementary Figure 7. Variation in protein levels of proliferative HT29NIS cells in normoxic conditions (NP), proliferative HT29NIS cells in hypoxic conditions (HP), quiescent HT29NIS cells in normoxic conditions (QP) and quiescent HT29NIS cells in hypoxic conditions (HQ). (A) Heat map of significant changes of protein levels when HP and HQ are compared. Green indicates higher levels and red indicates lower levels. (B) Venn diagram of significantly up-regulated proteins. (C) Venn diagram of significantly down-regulated proteins. A value of p <0.05 was considered significant. A value of p <0.05 was considered significant.

## Slide 8
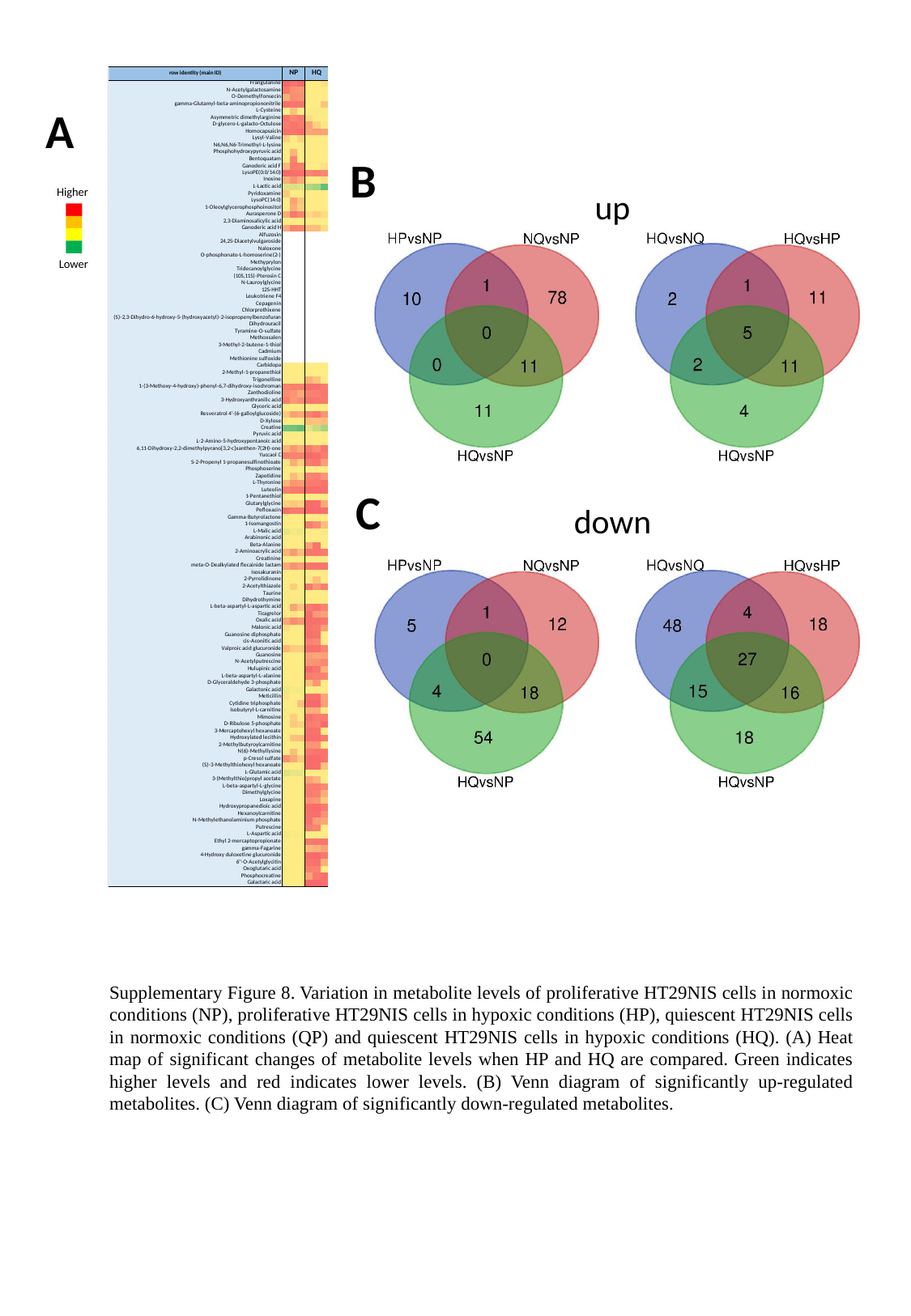

A
B
Higher
up
Lower
C
down
Supplementary Figure 8. Variation in metabolite levels of proliferative HT29NIS cells in normoxic conditions (NP), proliferative HT29NIS cells in hypoxic conditions (HP), quiescent HT29NIS cells in normoxic conditions (QP) and quiescent HT29NIS cells in hypoxic conditions (HQ). (A) Heat map of significant changes of metabolite levels when HP and HQ are compared. Green indicates higher levels and red indicates lower levels. (B) Venn diagram of significantly up-regulated metabolites. (C) Venn diagram of significantly down-regulated metabolites.

## Slide 9
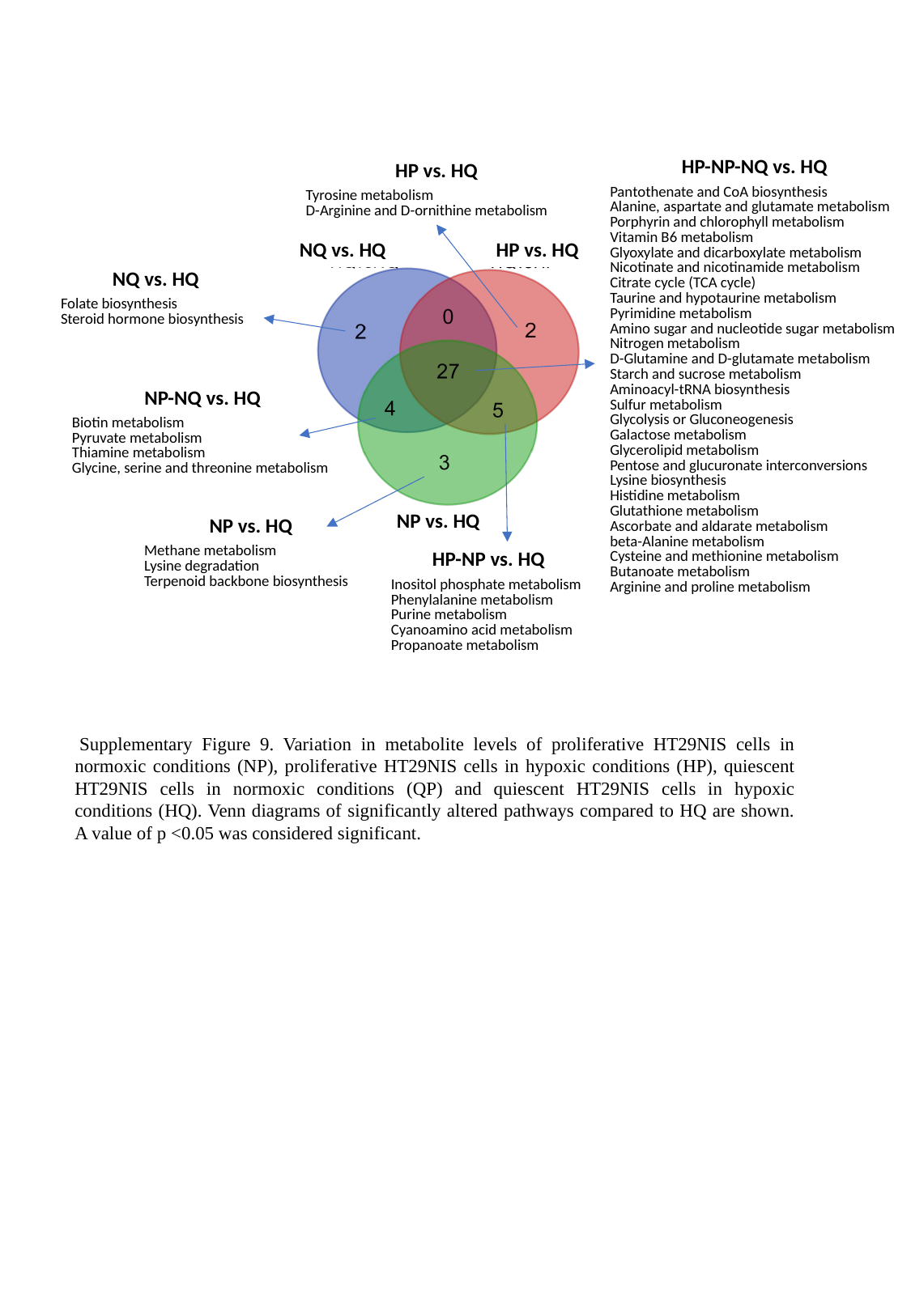

HP-NP-NQ vs. HQ
Pantothenate and CoA biosynthesis
Alanine, aspartate and glutamate metabolism
Porphyrin and chlorophyll metabolism
Vitamin B6 metabolism
Glyoxylate and dicarboxylate metabolism
Nicotinate and nicotinamide metabolism
Citrate cycle (TCA cycle)
Taurine and hypotaurine metabolism
Pyrimidine metabolism
Amino sugar and nucleotide sugar metabolism
Nitrogen metabolism
D-Glutamine and D-glutamate metabolism
Starch and sucrose metabolism
Aminoacyl-tRNA biosynthesis
Sulfur metabolism
Glycolysis or Gluconeogenesis
Galactose metabolism
Glycerolipid metabolism
Pentose and glucuronate interconversions
Lysine biosynthesis
Histidine metabolism
Glutathione metabolism
Ascorbate and aldarate metabolism
beta-Alanine metabolism
Cysteine and methionine metabolism
Butanoate metabolism
Arginine and proline metabolism
HP vs. HQ
Tyrosine metabolism
D-Arginine and D-ornithine metabolism
NQ vs. HQ
HP vs. HQ
NQ vs. HQ
Folate biosynthesis
Steroid hormone biosynthesis
NP-NQ vs. HQ
Biotin metabolism
Pyruvate metabolism
Thiamine metabolism
Glycine, serine and threonine metabolism
NP vs. HQ
NP vs. HQ
Methane metabolism
Lysine degradation
Terpenoid backbone biosynthesis
HP-NP vs. HQ
Inositol phosphate metabolism
Phenylalanine metabolism
Purine metabolism
Cyanoamino acid metabolism
Propanoate metabolism
 Supplementary Figure 9. Variation in metabolite levels of proliferative HT29NIS cells in normoxic conditions (NP), proliferative HT29NIS cells in hypoxic conditions (HP), quiescent HT29NIS cells in normoxic conditions (QP) and quiescent HT29NIS cells in hypoxic conditions (HQ). Venn diagrams of significantly altered pathways compared to HQ are shown. A value of p <0.05 was considered significant.
