# Supplementary material for: Tumor microenvironment affects exogenous sodium/iodide symporter expression
Source: Transl Oncol. 2020 Nov 17;14(1):100937. doi: 10.1016/j.tranon.2020.100937 (PMC7679261; doi:10.1016/j.tranon.2020.100937)
Supplement: Supplementary file 2 [file mmc2.docx]

**Supplementary methods**

**Proteomic analysis**

Cells were lysed in 10 mM tris-HCl, 150 mM NaCl, 1 mM EDTA, 1% Triton X-100, 0.1% SDS and protease Inhibitors (cOmplete™, Mini Protease Inhibitor Cocktail Tablets). After a 20-minute incubation at 4°C, cell extracts were centrifugated (16000 xg for 1 minutes at 4°C). Supernatants were collected, protein concentrations were measured and stored at -80 °C until proteomic analysis. Cell lysates (100 µg) were separated on 10 % SDS-PAGE gels, stained by InstantBlue (Expedeon) and gel containing the whole proteome were cut into ≈ 1 mm^3^ cubes. All reagents for protein digestion were freshly prepared in 50 mM NH_4_HCO_3_. Gel cubes were destained with 50% acetonitrile (ACN), reduced with 25 mM DTT at 56°C for 10 minutes, alkylated with 55 mM iodoacetamide at room temperature for 10 minutes in the dark, dehydrated with ACN and dried in a SpeedVac (Savant, Thermo). Gel pieces were rehydrated in 50 - 100 µL of trypsin (0.025 µg per µg of protein to digest) in 50 mM NH_4_HCO_3_ containing 0.01% ProteaseMAX surfactant (Promega), a trypsin enhancer, followed by a 4-hour incubation at 37 °C. After protein digestion, the resulting peptide extracts were separated from gel cubes. Formic acid was added to a final concentration of 5% and peptide extracts were centrifuged at 15000 xg for 10 minutes to remove degraded ProteaseMAX, dried in a SpeedVac, resuspended in ACN/H_2_O: 20/80 and stored at -20°C until LC-MS analysis.

The resulting peptides (10 μL) were analyzed using an ESI-Q Exactive Plus mass spectrometer (Thermo Fisher Scientific) incorporating a high-field Orbitrap analyser and coupled to an Ultimate 3000 RSL Capillary LC System (Thermo Fisher Scientific) as previously described {Duarte, 2013 #5004}. The system was set up for pre-concentration mode using a 300 μm x 5 mm trap column in back-flush configuration at 40°C (P/N 6720.0315). An EASY-Spray 15 cm x 150 μm column (P/N ES806) was connected to the system and coupled to the system with an EASY-Spray source (P/N ES081) operating at 40°C. The flow rate was 1.2 μl/min with a 5-45% gradient of solvent B (80% acetonitrile, 20% water, 0.1% formic acid) against solvent A (0.1% formic acid, 100% water) for 180 min. A top15 data-dependent method was used for MS/MS spectrum acquisition. Full-scan mass spectra were measured from 350 to 1500 m/z with an Automatic Gain Control Target set at 3x10^6^ ions and a resolution of 70,000. MS/MS scan was initiated at a resolution of 17,500 for ions with potential charge of 2+, 3+ and 4+ with a dynamic exclusion of 20 s. MS/MS were recorded with an Automatic Gain Control Target set at 5x10^4^ ions.

All MS raw data files were analyzed by Proteome Discoverer software 2.1.1.21 (Thermo Fisher, France) using the Sequest HT search engine against a database of protein sequences (Uniprot version2015_2). X corr confidence was held upper to 0.7 for all parameters. Precursor mass tolerance was set to 10 ppm and fragment ion tolerance was 0.02 Da with permission of two missed cleavages in the trypsin digests. A decoy database search strategy was also used to estimate the false discovery rate (FDR) to ensure the reliability of the proteins identified: a 1% target FDR as strict criteria and 5% target FDR as relaxed criteria using Percolator and at least two peptides were required for matching a protein entry for its identifications. The strict maximum parsimony principle was performed, and only peptide spectra with at least medium confidence were considered for protein grouping. Carbamidomethylation on cysteine was set as the static modification and oxidized methionine as the dynamic modification. For relative quantitation, both unique and razor peptides were considered to be a highly confident identification and used for quantification. Reporter ion abundances were corrected for isotopic impurities based on the manufacturer’s data sheets. Signal-to-noise (S/N) values were used to represent the reporter ion abundance with a co-isolation threshold of 75% and an average reporter S/N threshold of 10 and above required for quantitation spectra to be used. The S/N values of peptides, which were summed from the S/N values of the PSMs, were summed to represent the abundance of the proteins. The quantitative protein ratios were calculated and normalized by total peptide amount and scaled on channel average for each sample. All experiments were performed in four independent replicates. Proteins between groups were considered significantly different when p ≤ 0.05, up-regulated when fold change ≥2 and down-regulated when fold change ≤ 0.5. The IPA tool (Ingenuity Systems, Redwood City, CA, [www.qiagen.com/ingenuity](http://www.qiagen.com/ingenuity)) to analyze networks of differentially expressed proteins.

**Metabolomics analysis**

After appropriate incubation, cells were quickly rinsed twice with a cold sucrose solution (150 mM), and cold methanol (HPLC grade, Merck Millipore, Billerica, MA, USA) was added to each well. Plates were incubated overnight at -20 °C and then centrifuged at 15000 g for 15 minutes. Supernatants were removed and dried using a SpeedVac concentrator (SVC100H, SAVANT, Thermo Fisher Scientific, Illkirch, France). Lyophilized samples were resuspended in 50 µl of a 20:80 acetonitrile-H_2_O mixture (HPLC grade, Merck Millipore) prior to LC-MS/MS analyses.

Metabolic profiling was performed using LC-MS/MS. Liquid chromatography analyses were performed using the DIONEX Ultimate 3000 HPLC system (Thermo Fisher Scientific, Illkirch, France). Ten microliters of each sample were injected onto a Synergi 4 µm Hydro-RP 80 A 250 x 3.0 mm column (Phenomenex, Le Pecq, France). Mobile phases comprised 0.1 % formic acid (Thermo Fisher Scientific) in water (A) and 0.1 % formic acid in acetonitrile (B). The following gradient was established, with a flow rate of 0.9 ml/min: 0 % mobile phase B from 0 to 5 min, 0 – 95 % B from 5 to 21 min, holding at 95 % B to 21.5 min, 95 – 0 % B from 21.5 to 22 min, and holding at 0 % B until 25 min for column equilibration. The mass spectrometry analysis was performed on a Q Exactive Plus Orbitrap mass spectrometer (Thermo Fisher Scientific) with a heated electrospray ionization source (HESI II) operating in both positive and negative mode. High-resolution accurate mass (HRAM) full-scan MS and the top 5 MS/MS spectra were collected in data-dependent mode at resolving powers of 70000 and 35000, respectively. A quality control (QC) sample was prepared from an equal mixture of all collected samples. The QC sample was injected at the beginning of the run and after every 9 samples to monitor the stability of the mass spectrometer performance.

Raw data files were converted to MZxml files using MSconvert (version 2.1, ProteoWizard) {Holman, 2014 #7898}. The data obtained from positive and negative ionization modes were analyzed separately using MZmine (version 2.29) {Pluskal, 2010 #7887}. Isolated chromatograms were generated for each mass, with a noise threshold of 105. A local minimum search algorithm was used to select the validated peaks. Peaks were then aligned by a random sample consensus (RANSAC) algorithm with a tolerance of 10 ppm for m/z and 1 min for the retention time. Missing values were added using the same m/z and RT ranges observed in detected samples, when possible. Peaks were then identified using the human metabolome database (HMDB, version 3.0) with a 15 ppm mass tolerance {Wishart, 2013 #7891}. Only identified metabolites were retained for further pathway analyses. The results obtained with each polarity were combined, and for metabolites that were identified in both modes, we retained the metabolites with the higher mean intensity values and observed in most samples. Metabolites between groups were considered significantly different when p ≤ 0.05, up-regulated when fold change ≥ 2 and down-regulated when fold change ≤ 0.5. Pathway analysis was performed using MetaboAnalyst {Chong, 2018 #8864 ; Chong, 2019 #8865} (https://www.metaboanalyst.ca/).
